# Synthesis of Novel Methyl 3-(hetero)arylthieno[3,2-*b*]pyridine-2-carboxylates and Antitumor Activity Evaluation: Studies In Vitro and In Ovo Grafts of Chick Chorioallantoic Membrane (CAM) with a Triple Negative Breast Cancer Cell Line

**DOI:** 10.3390/molecules26061594

**Published:** 2021-03-13

**Authors:** Bruna R. Silva, Rita Rebelo, Juliana M. Rodrigues, Cristina P. R. Xavier, M. Helena Vasconcelos, Maria-João R. P. Queiroz

**Affiliations:** 1 Centre of Chemistry, University of Minho, 4710-057 Braga, Portugal; brunadansilva1967@gmail.com (B.R.S.); id7018@alunos.uminho.pt (J.M.R.); 2 Cancer Drug Resistance Group, IPATIMUP—Institute of Molecular Pathology and Immunology, University of Porto, 4200-135 Porto, Portugal; rrebelo@ipatimup.pt (R.R.); hvasconcelos@ipatimup.pt (M.H.V.); 3 i3S—Instituto de Investigação e Inovação em Saúde, Universidade do Porto, 4200-135 Porto, Portugal; 4 Department of Biological Sciences, FFUP—Faculty of Pharmacy, University of Porto, 4050-313 Porto, Portugal

**Keywords:** anticancer activity, Suzuki-Miyaura coupling, thieno[3,2-*b*]pyridines, triple negative breast cancer

## Abstract

A series of novel functionalized methyl 3-(hetero)arylthieno[3,2-*b*]pyridine-2-carboxylates **2a**–**2h** were synthesized by C-C Pd-catalyzed Suzuki-Miyaura cross-coupling of methyl 3-bromothieno[3,2-*b*]pyridine-2-carboxylate with (hetero)aryl pinacol boranes, trifluoro potassium boronate salts or boronic acids. Their antitumoral potential was evaluated in two triple negative breast cancer (TNBC) cell lines—MDA-MB-231 and MDA-MB-468, by sulforhodamine B assay. Their effects on the non-tumorigenic MCF-12A cells were also evaluated. The results demonstrated that three compounds caused growth inhibition in both TNBC cell lines, with little or no effect against the non-tumorigenic cells. The most promising compound was further studied concerning possible effects on cell viability (by trypan blue exclusion assay), cell proliferation (by bromodeoxyuridine assay) and cell cycle profile (by flow cytometry). The results demonstrated that the GI_50_ concentration of compound **2e** (13 μM) caused a decreased in MDA-MB-231 cell number, which was correlated with a decreased in the % of proliferating cells. Moreover, this compound increased G0/G1 phase and decreased S phases, when compared to control cells (although was not statistic significant). Interestingly, compound **2e** also reduced tumor size using an in ovo CAM (chick chorioallantoic membrane) model. This work highlights the potential antitumor effect of a novel methyl 3-arylthieno[3,2-*b*]pyridine-2-carboxylate derivative.

## 1. Introduction

Regardless the great number of research studies worldwide to improve our understanding and effort to develop new therapies, breast cancer is still the most frequent diagnosed cancer and the main cause of cancer death in women [1]. Nowadays, due to a recent histopathologic classification, breast cancer is divided into different subtypes depending on the expression of key proteins such as human epidermal growth factor receptor 2 (HER-2) estrogen and progesterone receptors; Ki67; claudins; epidermal growth factor receptor (EGFR) and others. Thus, the subtypes of breast cancer are designated as luminal A, luminal B, HER2-enriched (+), basal-like and claudin-low [2]. The most aggressive, invasive and with poor prognosis type of breast cancer is the triple negative breast cancer (TNBC), which is immunohistochemically characterized by the lack of estrogen receptor, progesterone receptor and absence of HER2 amplification [1]. TNBC accounts for 15 to 20% of breast cancer cases and is a highly heterogenic and aggressive type of cancer, with patients having better response to neoadjuvant therapy [3]. Although the knowledge about breast cancer has considerably evolved in the last years, no advances have been made regarding TNBC treatment, thus chemotherapy remains the standard therapeutic approach for all stages of TNBC [4]. For that reason, the discovery of novel drugs is crucial to find new therapeutic options for treatment of TNBC. 

Over the last years, several thienopyridine derivatives have been synthesized based on the high probability of having different biological properties, namely for cancer treatment. In 2004, Munchhof et al. reported the synthesis of substituted 7-(indol-5-yl)aminothieno [3,2-*b*]pyridines and their potential as inhibitors of the vascular endothelial growth factor receptor 2 (VEGFR-2), which is a receptor of tyrosine kinase that triggers signalization pathways that cause endothelial cell proliferation and tumor vascularization (angiogenesis) [5]. Boschelli et al. reported in 2005 the synthesis of 2-heteroaryl substituted 7-[(2,4-dichloro-5-methoxyphenyl)amino]thieno [3,2-*b*]pyridine-6-carbonitriles, which act by inhibiting the non-receptor tyrosine kinase Src, a kinase implicated in cancer, osteoporosis and ischemic diseases when is overexpressed or overactivated [6]. In 2008, Claridge et al. showed that substituted 7-arylethers of thieno[3,2-*b*]pyridine phenylacetylthioureas inhibit VEGFR-2 and c-Met (receptor for hepatocyte growth factor) both implicated in angiogenesis acting synergistically [7].

Since 2010, both research groups involved in this work have been interested in the synthesis and antitumor evaluation of new functionalized thieno[3,2-*b*]pyridines, either on the pyridine ring by C-C Suzuki-Miyaura [8], Sonogashira [9], C-N Buchwald-Hartwig and C-O Ullmann [10] couplings, or on the thiophene by C-N coupling [11] and C-C Sonogashira followed by intramolecular cyclization [12]. Some of the compounds synthesized in the referred works showed GI_50_ values below 10 µM in different human tumor cell lines and for the most promising compounds, some insights on the mechanism of action were assessed, namely, the effects in the cell cycle of NCI-H460 cells by flow cytometry and in apoptosis using Annexin(V)-PI.

Queiroz et al. later reported the synthesis and biological evaluation of novel 1-aryl-3-[3-(thieno[3,2-*b*]pyridin-7-ylthio)phenyl]ureas with hydrophobic groups *m*- or *p*-F, *m*-CH3, *m*-CF3, and *m*-CF3/*p*-Cl (substitution pattern of Sorafenib) relative to the urea moiety in the terminal aryl ring, as antiangiogenic compounds using several assays in Human Umbilical Endothelial Cells (HUVEC) [13]. Compounds bearing *m*-F, *m*-CH_3_ and *m*-CF_3_**/***p*-Cl showed also antitumor potential against two different human breast cancer cell lines, MCF-7 (Estrogen receptor positive, ER+) and MDA-MB-231 (TNBC). These compounds also showed very low cytotoxicity in the MCF-10A cell line from non-tumorigenic mammary epithelial cells [14].

In summary, some thieno[3,2-*b*]pyridine derivatives have already demonstrated their potential as anticancer and/or antiangiogenic agents. 

Herein the synthesis of novel methyl 3-(hetero)arylthieno[3,2-*b*]pyridine-2-carboxylates by C-C Suzuki Miyaura cross-coupling is presented. Their antitumor potential was further evaluated in two different TNBC cell lines. Three of the thieno[3,2-*b*]pyridine derivatives presented antitumor activity against the TNBC MDA-MB-231 and MDA-MD-468 cells, without showing relevant toxicity against a non-tumorigenic mammary epithelial cell line (MCF-12A). Moreover, compound **2e** significantly decreased the number of viable cells and cellular proliferation of MDA-MB-231 cells, as well as, increased G0/G1 phase and decreased S phase (although not statistically significant). Importantly, compound **2e** also decreased the size of in ovo grafts of chick chorioallantoic membrane (CAM) with MDA-MD-231 cells. Thus, this work points to the antitumor potential of a series of novel thienopyridine derivatives, highlighting one compound as a promising molecule against TNBC.

## 2. Results

### 2.1. Synthesis of Methyl 3-(hetero)arylthieno[*3,2*-b]pyridine-2-carboxylates **2a**–**2h**

The precursor methyl 3-bromothieno[3,2-*b*]pyridine-2-carboxylate **1** was prepared according to a known procedure, from the corresponding 3-amino compound [15] using *t*-BuONO and CuBr_2_ [12], and it was reacted with several commercial (hetero)aryl pinacolboranes, trifluoro potassium boron salts or boronic acids under Pd-catalyzed Suzuki-Miyaura [8,16,17,18] cross-coupling conditions (1.2–1.6 equiv. of the boronated compound, 2–4 mol% of Pd(dppf).CH_2_Cl_2_ (1:1), 6 equiv. of K_2_CO_3_ in DME:H_2_O (3:1) at 100 °C for 3–4.5 h) to give the methyl 3-(hetero)arylthieno[3,2-*b*]pyridine-2-carboxylates **2a**–**2h** in moderate to high yields (35–84%) after column chromatography (Table 1). Compounds **2a**–**2h** were prepared with no substituents, with electron donating groups—EDGs—(OMe, Me) and electron withdrawing groups—EWGs—(Cl, CN, CF_3_) in the *para*-position of the phenyl ring relative to the C-C bond formed or with an electron-deficient ring (pyridine) or an electron-rich one (furan).

Having compounds **2a**–**2h** in hands their potential as antitumor compounds was evaluated.

### 2.2. Cell Growth Inhibitory Effect of the Synthesized Compounds on Two Different TNBC Cell Lines

The antitumor potential of the synthesized compounds **2a**–**2h** was evaluated in vitro using the sulforhodamine B (SRB) assay [19,20], which allows to determine the GI_50_ concentration of each compound, i.e., the drug concentration that inhibits 50% of the cell growth. Thus, the compounds were tested in two different triple negative breast cancer (TNBC) cells, MDA-MB-231 and MDA-MB-468. Unfortunately, it was not possible to determine the GI_50_ values for some of the compounds synthesized, since these compounds formed crystals in the culture medium at higher concentrations. Therefore, in these cases, the GI_50_ values were considered higher than the maximum concentration tested without crystal formation. Results presented in Table 2 demonstrated that three of the synthesized compounds (**2e**, **2f**, **2h**) tested significantly inhibited the growth of both TNBC cell lines with a GI_50_ relatively low. Indeed, compound **2h** presented the lowest GI_50_ value in the MDA-MB-468 TNBC cell line. Even though it was not possible to establish structure-activity relationships (SARs), the furan derivative **2h**, with an electron-rich ring, seems more selective for the MDA-MB-468 cell line. Moreover, the same was observed for compound **2f** with an EWG (*p*-CN). Taken together compounds **2e**, **2f** and **2h**, showed promising anticancer activity against TNBC cell lines.

### 2.3. Evaluation of the Cytotoxicity of the Most Promising Compounds against a Non-Tumorigenic Cell Line 

The most promising compounds **2e**, **2f** and **2h**, were then evaluated regarding their toxicity against the non-tumorigenic mammary epithelial MCF-12A cell line. For that, the SRB assay was performed using the GI_50_ concentrations of each of the three compounds obtained in the corresponding TNBC cell lines. The results (Table 3) demonstrated that compounds **2e**, **2f** and **2h**, at the GI_50_ concentrations previously obtained in the most sensitive TNBC cancer cell line (Table 2), caused a small or no effect in the growth of the non-tumorigenic MCF-12A cells. These results suggest that the three compounds are selective for cancer cells, not affecting the non-tumorigenic cell line, at least at the concentrations tested. It should be noted that the duration of the SRB assay carried out in the non-tumorigenic cells is longer than the SRB assay carried out in cancer cells, since the non-tumorigenic cells have a slower cell growth rate. This longer duration of the assay allows evaluation of possible delayed toxic effects of the compounds in the non-tumorigenic cells. 

### 2.4. Effect of Compound ***2e*** on Number of Viable Cells and on the Proliferation of TNBC MDA-MB-231 Cells

Compound **2e** has the particularity of having part of its hydrophobic tail (the phenyl ring bearing a Cl atom) similar to Sorafenib [21], which is an antiangiogenic drug known as an active multikinase inhibitor, currently approved for the treatment of different types of cancers [22]. However, Sorafenib has not been included in the clinical practice for breast cancer treatment given its high toxicity [23,24]. 

**Figure 1:**
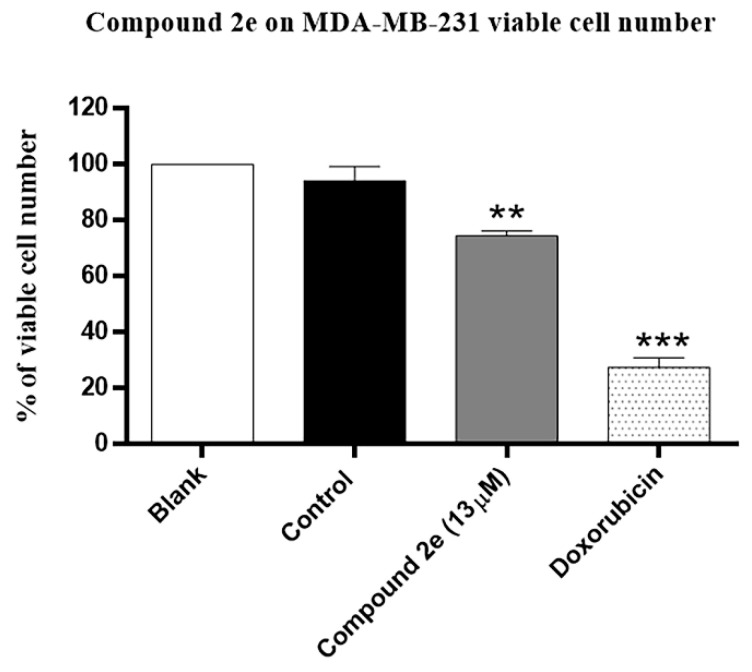
Effect of compound **2e** on MDA-MB-231 viable cell number, determined by trypan blue exclusion assay. Cells were treated for 48 h with medium (Blank), control (DMSO; vehicle), compound **2e** (at 13 µM) and Doxorubicin (positive control; 50 nM). Results represent the mean ± S.E.M. of at least three independent experiments. ** *p* ≤ 0.01 and *** *p* ≤ 0.001 of Control vs. Treatments.

Therefore, we decided to conduct some preliminary activity studies on compound **2e**. Its effect on TNBC viable cell number and cell proliferation was assessed after 48 h treatment, using the trypan blue exclusion assay and the bromodeoxyuridine (BrdU) incorporation assay, respectively. For that, the MDA-MB-231 cell line was treated with the GI_50_ concentration of compound **2e**, which corresponds to 13 μM, with the vehicle DMSO (control; at the same concentration used for the compound), and doxorubicin, used as positive control. The obtained results demonstrated that as expected, doxorubicin significantly reduced MDA-MB-231 viable cell number. In addition, the GI_50_ concentration of compound **2e** significantly reduced MDA-MB-231 viable cell number in comparison with the control treatment (Figure 1).

**Figure 2:**
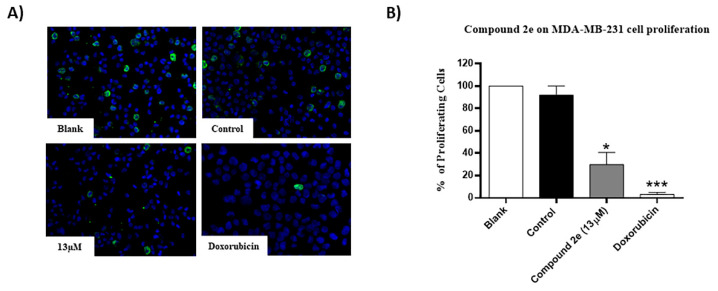
Effect of compound **2e** on MDA-MB-231 cellular proliferation, determined by bromodeoxyuridine (BrdU) incorporation assay. Cells were treated for 48 h with medium (Blank), control (DMSO; vehicle), compound **2e** (at 13 µM) and Doxorubicin (positive control; 50 nM). (**A**) Representative fluorescence microscopy images of BrdU incorporation (green) and DAPI stained nuclei (blue). Amplification = 200×. (**B**) Percentage of BrdU-incorporating cells. The results are presented as the mean ± S.E.M. of three independent experiments. * *p* ≤ 0.05 and *** *p* ≤ 0.001 of Control vs. Treatments.

Moreover, as expected, doxorubicin significantly reduced MDA-MB-231 cellular proliferation. Furthermore, the GI_50_ concentration of compound **2e** also significantly decreased the % of proliferating MDA-MB-231 cells, when compared to the control (Figure 2). These results suggested that compound **2e** interferes with the TNBC viable cell number and proliferation.

### 2.5. Effect of Compound ***2e*** on Cell Cycle Profile in TNBC MDA-MB-231 Cells

**Figure 3:**
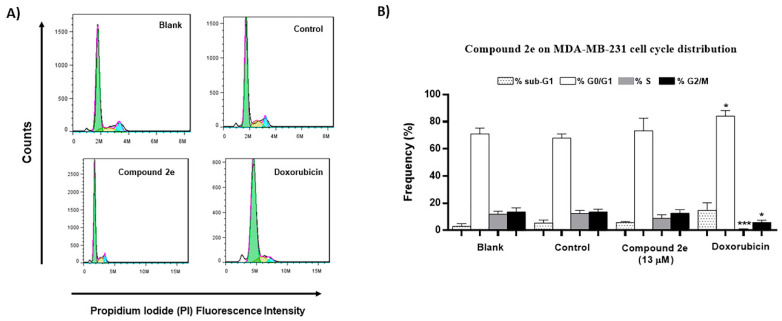
Effect of compound **2e** on MDA-MB-231 cell cycle distribution, analyzed by flow cytometry following incubation with propidium iodide (PI). Cells were treated for 48 h with medium (Blank), control (DMSO; vehicle), compound **2e** (at 13 µM) and Doxorubicin (positive control; 50 nM). (**A**) Representative histograms of the MDA-M-231 cell cycle profile with the different treatments. (**B**) Percentage of MDA-MB-231 cells in the different phases of the cell cycle. Results represent the mean ± S.E.M. of at least three independent experiments. * *p* ≤ 0.05 and *** *p* ≤ 0.001 of Control vs. Treatments.

Considering the previous results, we further analyzed the possible effect of compound **2e** on TNBC MDA-MB-231 cell cycle profile, assessed after 48 h treatment, using the flow cytometry analysis with propidium iodide (PI). For that, the MDA-MB-231 cell line was treated with 13 μM of compound **2e** (GI_50_ concentration), the vehicle DMSO (control) and doxorubicin. As predictable, doxorubicin (used as positive control) significantly increased the G0/G1 phase and decreased S and G2/M phases in the MDA-MB-231 cells. The results demonstrated that the GI_50_ concentration of compound **2e**, although not statistically significant, increased G0/G1 phase and decreased S phase, when compared to control cells (Figure 3). These results suggested that compound **2e** interferes with the cell cycle profile of TNBC MDA-MB-231 cells.

### 2.6. Antitumor Effect of Compound ***2e*** *in Ovo* Grafted with MDA-MB-231 Cells Using the Chick Chorioallantoic Membrane (CAM) Assay 

The above results indicate that compound **2e** is the most promising compound against the MDA-MD-231 cell line. In addition, this compound decreased the number of viable and proliferating tumor cells. Since compound **2e** has a structural similarity with the antiangiogenic drug Sorafenib, the study of the antiangiogenic and antitumor potential of this compound was attempted using the chick embryo chorioallantoic membrane (CAM) assay [25]. Thus, CAM assay was performed by grafting TNBC MDA-MB-231 cells in ovo, in order to create tumors in the eggs, which was followed by testing the effect of the GI_50_ concentration (13 µM) of compound **2e** on tumor formation.

**Figure 4:**
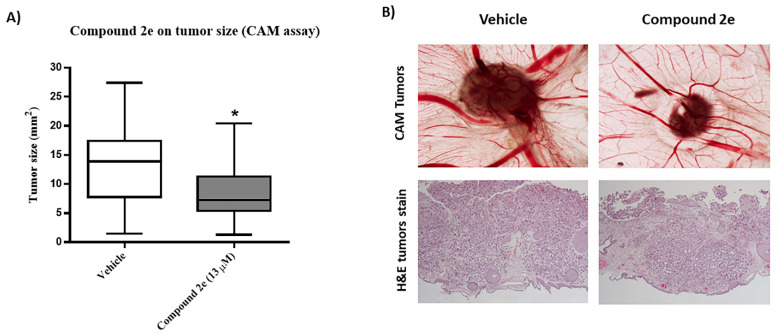
Effect of compound **2e** on tumor size, using the in ovo chick embryo chorioallantoic membrane (CAM) model. TNBC MDA-MB-231 cells were grafted in the chicken eggs, and after 24 h were treated with the vehicle (DMSO) or with the compound **2e** at 13 μM, for 48 h. (**A**) Tumor size of eggs treated with vehicle and compound **2e**. Data is from two independent experiments. * *p* ≤ 0.05 of Vehicle vs. Treatment. (**B**) Representative images (1.25× amplification) or Hematoxylin & Eosin (H&E) stain of tumor size of cells treated with vehicle (DMSO) and compound **2e**.

Results showed that (Figure 4A) treatment of grafted eggs for 48 h with the GI_50_ concentration of compound **2e** efficiently decreased tumor formation (tumor size), when compared with the control group (vehicle). Unfortunately, the antiangiogenic potential was not possible to analyze due to the high vascularization induced by breast cancer cells, which led to some degree of inflammation. The reduction in tumor size was well visible (Figure 4B). In addition, using hematoxylin and eosin (H&E) staining, viability of tumors at both conditions was confirmed (Figure 4B). Remarkably, the CAM assay confirmed a promising anti-tumorigenic effect of compound **2e**, although no information regarding the antiangiogenic potential was possible to obtain.

## 3. Discussion

From the eight methyl 3-(hetero)arylthieno[3,2-*b*]pyridine-2-carboxylates **2a**-**2h** synthesized (Table 1), the compounds obtained with the best yields were **2a** (Ph), **2b** (*p*-Me), **2c** (*p*-OMe) without substitution or with EDGs, and **2e** (*p*-Cl). Although for phenyl rings bearing EWGs (*p*-CF_3_ and *p*-CN) the yields were only moderate, for compound **2e** the yield was excellent (82%) may be due to the positive mesomeric effect of the chlorine. Despite the different electron character of the heterocyclic rings, pyridine and furan, the resulting compounds **2g** and **2h** were isolated in good yields.

The evaluation of the effect of this series of compounds on cell growth (by the SRB assay) allowed to find the three most promising compounds, namely compounds **2e**, **2f** and **2h**, which under the concentrations tested showed antitumor effect in vitro against the TNBC cell lines. Importantly, the three compounds had little or no effect against the non-tumorigenic cancer cell line MCF-12A, at the concentrations tested (which inhibited the growth of the cancer cells by 50%).

Since compound **2e** has the particularity of having part of its hydrophobic tail (the phenyl ring coupled to a *p*-Cl atom) similar to Sorafenib, an approved anticancer agent for other cancer types [21], it was decided to conduct preliminary studies on the antitumor activity of this novel compound. The results demonstrated that compound **2e** decreased viable cell number and cell proliferation in the TNBC MDA-MB-231 cells. Interestingly, compound **2e** did not cause alterations in the levels of two important apoptotic markers, PARP or caspase-3, therefore suggesting that apoptosis is not involved in the mechanism of action of this compound (data not shown). Remarkable, this work showed that compound **2e** reduced tumor size of grafted MDA-MB-231 cells in ovo CAM model. The CAM assay allows to study the effect of compounds on the angiogenesis process, as well as to evaluate the effect on tumor growth (tumorigenic response), being suitable as an in vivo model for the screening of potential novel drugs, as an alternative to mouse models [26]. 

Interestingly, studies conducted in TNBC cells demonstrated that Sorafenib is able to inhibit cell proliferation and tumor growth [27,28,29]. Similar effects were observed in the present study, with MDA-MB-231 cells treated with compound **2e**, both in vitro and in ovo. It is possible that the hydrophobic tail might be partly responsible for the anticancer activity of these molecules, but this hypothesis requires evaluation. Further studies should be conducted to better understand the mechanism of action of compound **2e**.

## 4. Materials and Methods

### 4.1. Synthesis

Melting points (°C) were determined in a SMP3 Stuart apparatus. ^1^H, ^13^C and ^19^F NMR spectra were recorded on a Bruker Advance III (Bruker, Bremen, Germany) at 400, 100.6 and 376.48 MHz respectively (see Suppl. Materials), using the signals of the non-deuterated solvents of CHCl_3_ (7.27 ppm) of the CDCl_3_ or of DMSO (2.49 ppm) of the DMSO-*d*_6_, as internal reference relatively to TMS (0 ppm). DEPT (θ = 135°) and bi-dimensional homo ^1^H–^1^H (COSY) and heteronuclear correlations ^1^H-^13^C (HMQC and HMBC) were used to attribute some signals (see Supplementary Materials). Elemental analysis was performed on a LECO CHNS 932 elemental analyzer (LECO Corporation, Michigan, USA). The low-resolution MS spectra were obtained by ESI [M + H]^+^ using a Thermo-Finnigan LXQ (Thermo Fisher Scientific, Waltham, MA, USA) at the CQUM. HRMS were obtained at the external service of mass spectrometry of the University of Vigo using EI (M^+^). Reactions were followed by thin layer chromatography (TLC). Column chromatography was performed on Macherey-Nagel silica gel 0.060–0.200 mm, 60 A, and dry flash chromatography on silica gel 0.035–0.070 mm, 60 A, to purify the compounds. Petroleum ether refers to the boiling range 40–60 °C. Ether refers to dietylether. DME refers to 1,2-dimethoxyethane. The catalyst PdCl_2_(dppf).CH_2_Cl_2_ (1:1) refers to 1,10-bis(diphenylphosphino)ferrocenedichloropalladium (II), complex with dichloromethane (1:1) and was purchased from Aldrich (Sigma-Aldrich, St. Louis, MO, USA).

#### 4.1.1. General Procedure for the Suzuki-Miyaura Cross-coupling Products **2a**–**h**

In a round flask, DME (3 mL) and water (1 mL), compound **1**, (het)aryl boronic acids, pinacol esters, or potassium trifluoroborates (1.2–1.6 equiv.), PdCl_2_ (dppf).CH_2_Cl_2_ (1:1) (2 mol% unless stated), K_2_CO_3_ (6 equiv.) were added and the mixture was heated with stirring at 100 °C for 3–4.5 h. The reactions were monitored by TLC. After cooling, water was added (10 mL) and extraction with ethyl acetate (3 × 10 mL) was performed. The organic phases were collected, dried (MgSO_4_), filtered and the solvent removal under reduced pressure gave a solid that was submitted to column chromatography or to dry flash using solvent gradient from neat petroleum ether to mixture of ether/petroleum ether, increasing 10% of ether each time, to give compounds **2a**–**2h**.

##### Methyl 3-phenylthieno[3,2-*b*]pyridine-2-carboxylate (**2a**)

From compound 1 (0.100 g, 0.370 mmol) and phenylboronic acid pinacol ester (0.0900 g, 0.440 mmol) following the general procedure for 3 h; purification by column chromatography using 50% ether/petroleum ether, gave compound 2a as a white solid (0.0730 g, 74%), m.p. 152–154 °C. ^1^H-NMR (DMSO-*d*_6_, 400MHz) δ = 3.75 (3H, s, OMe), 7.43–7.48 (5H, m, Ar-H), 7.56 (1H, dd, *J* = 8.0 and 4.4 Hz, 6-H), 8.61 (1H, dd, *J* = 8.0 and 1.6 Hz, 7-H), 8.74 (1H, dd, *J* = 4.4 and 1.6 Hz, 5-H) ppm. ^13^C-NMR (DMSO-d_6_, 100.6 MHz) δ = 52.6 (OMe), 122.0 (6-CH), 127.4 (2 × CH), 128.0 (CH), 130.5 (2 × CH), 131.0 (C), 132.0 (7-CH), 133.1 (C), 134.2(C), 142.6 (C), 148.6 (5-CH), 153.3 (C), 162.1 (C=O) ppm. MS (ESI) *m*/*z* (%): 270.03 [M + H]^+^ (100%). Elemental Anal. calcd for C_15_H_11_NO_2_S: C 66.89%, H 4.12%, N 5.20%, S 11.91%. Found: C 67.08%, H 4.09%, N 5.46%, S 12.16%.

##### Methyl 3-(*p*-tolyl)thieno[3,2-*b*]pyridine-2-carboxylate (**2b**)

From compound **1** (0.150 g, 0.554 mmol) and potassium *p*-tolyltrifluoroborate (0.132 g, 0.664 mmol) and heating for 4.5 h, after purification by column chromatography using 40% ether/petroleum ether, compound 2b was obtained as a white solid (0.132 g, 84%), m.p. 153–154 °C. ^1^H-NMR (CDCl_3_, 400 MHz) δ = 2.44 (3H, s, Me), 3.85 (3H, s, OMe), 7.33 (2H, d, *J* = 8.0 Hz, 3′ and 5′-H), 7.39 (1H, dd, *J* = 8.4 and 4.8 Hz, 6-H), 7.42 (2H, d, *J* = 8.0 Hz, 2′ and 6′-H), 8.24 (1H, dd, *J* = 8.4 and 1.6 Hz, 7-H), 8.80 (1H, dd, *J* = 4.8 and 1.6 Hz, 5-H) ppm. ^13^C-NMR (CDCl_3_, 100.6 MHz) δ = 21.5 (Me), 52.4 (OMe), 121.1 (6-CH), 128.7 (3′ and 5′-CH), 130.1 (2′ and 6′-CH), 130.12 (C), 130.7 (7-CH), 131.2 (C), 134.9 (C), 138.2 (C), 144.0 (C), 148.5 (5-CH), 154.6 (C), 162.7 (C=O) ppm. MS (ESI) *m*/*z* (%): 284.07 [M + H]^+^ (83%). Elemental Anal. calcd for C_16_H_13_NO_2_S: C 67.82%, H 4.62%, N 4.94%, S 11.32%. Found: C 68.15, H 4.35, N 5.20, S 11.69%.

##### Methyl 3-(4-methoxyphenyl)thieno[3,2-*b*]pyridine-2-carboxylate (**2c**)

From compound **1** (0.100 g, 0.370 mmol) and potassium 4-methoxyphenyltrifluoroborate (0.094 g, 0.440 mmol), reacting for 4 h, after purification by column chromatography using 25% ether/petroleum ether, compound **2c** was obtained as a white solid (0.0770 g, 70%), m.p. 190–191 °C. ^1^H-NMR (CDCl_3_, 400MHz) δ = 3.85 (3H, s, OMe), 3.89 (3H, s, OMe), 7.05 (2H, d, *J* = 8.8 Hz, 3′ and 5′-H), 7.39 (1H, dd, *J* = 8.0 and 4.4 Hz, 6-H), 7.50 (2H, d, *J* = 8.8 Hz, 2′ and 6′-H), 8.23 (1H, dd, *J* = 8.0 and 1.6 Hz, 7-H), 8.80 (1H, dd, *J* = 4.4 and 1.6 Hz, 5-H) ppm. ^13^C-NMR (CDCl_3_, 100.6 MHz) δ = 52.5 (OMe), 55.2 (OMe), 113.4 (3′ and 5′-CH), 121.1 (6-CH), 125.2 (C), 130.7 (7-CH), 130.9 (C), 131.6 (2′ and 6′-CH), 134.9 (C), 143.6 (C), 148.5 (5-CH), 154.2 (C), 159.7 (4′-C), 162.9 (C=O) ppm. MS (ESI) *m*/*z* (%): 300.06 [M + H]^+^ (100%). Elemental Anal. calcd for C_16_H_13_NO_3_S: C 64.20%, H 4.38%, N 4.68%, S 10.71%. Found: C 64.20%, H 4.45%, N 4.84%, S 10.33%.

##### Methyl 3-[4-(trifluoromethyl)phenyl]thieno[3,2-*b*]pyridine-2-carboxylate (**2d**)

From compound **1** (0.100 g, 0.370 mmol) and potassium 4-(trifluoromethyl)phenyltrifluoroborate (0.106 g, 0.440 mmol), reacting for 4 h, after purification by column chromatography using 40% ether/petroleum ether, compound 2d was obtained as a white solid. This was recrystallized from ether to give a colorless crystals (0.0350 g, 40%), m.p. 170–171 °C. ^1^H-NMR (CDCl_3_, 400MHz) δ = 3.85 (3H, s, OMe), 7.43 (1H, broad d, 6-H), 7.66 (2H, d, *J* = 8.0 Hz, 2′ and 6′-H), 7.78 (2H, d, *J* = 8.0 Hz, 3′ and 5′-H), 8.28 (1H, broad d, *J* = 8.0 Hz, 7-H), 8.80 (1H, broad s, 5-H) ppm. ^13^C-NMR (CDCl_3_, 100.6 MHz) δ = 52.7 (OMe), 120.6 (6-CH), 124.8 (q, *J* = 271.62 Hz, CF_3_), 124.9 (q, *J* = 3.6 Hz, 3′ and 5′-CH), 130.3 (q, *J* = 32.4 Hz, CCF3), 130.8 (2′ and 6′-CH), 131.0 (7-CH), 132.3 (C), 135.0 (C), 137.0 (C), 142.1 (C), 148.8 (5-CH), 153.3 (C), 162.4 (C=O) ppm. ^19^F-NMR (CDCl_3_, 376.48 MHz): δ = −62.63 (s, F_3_) ppm. MS (ESI) *m*/*z* (%): 338.05 [M + H]^+^ (100%). Elemental Anal. calcd for C_16_H_10_F_3_NO_2_S: C 56.97%, H 2.99%, N 4.15%, S 9.51%. Found: C 56.62%, H 2.65%, N 4.35%, S 9.81 %.

##### Methyl 3-(4-chlorophenyl)thieno[3,2-*b*]pyridine-2-carboxylate (**2e**)

From compound **1** (0.120 g, 0.443 mmol) and potassium (4-chlorophenyl)trifluoroborate (0.116 g, 0.532 mmol) reacting for 3 h, purification by column chromatography using 50% ether/petroleum ether, compound **2e** was obtined as a white solid (0.102 g, 82%), m.p. 195–196 °C. ^1^H-NMR (CDCl_3_, 400MHz) δ = 3.85 (3H, s, OMe), 7.43 (1H, dd, *J* = 8.4 and 4.4 Hz, 6-H), 7.49 (4H, coalesced doublets, Ar-H), 8.27 (1H, broad d, *J* = 8.4 Hz, 7-H), 8.81 (1H, broad s, 5-H) ppm. ^13^C-NMR (CDCl_3_, 100.6 MHz) δ = 52.6 (OMe), 121.3 (6-CH), 128.3 (2 × CH), 131.3 (7-CH), 131.7 (2 × CH), 132.2 (C), 134.7 (C), 135.1 (C), 142.1 (C), 148.4 (5-CH), 153.4 (C), 157.3 (C), 162.5 (C=O) ppm. HRMS (EI) Calcd. for C_15_H_10_^35^ClNO_2_S [M + ^35^Cl] 303.0121; found: 303.0104. Calcd. for C_15_H_10_^37^ClNO_2_S [M+^37^Cl] 305.0091; found: 305.0099.

##### Methyl 3-(4-cyanophenyl)thieno[3,2-*b*]pyridine-2-carboxylate (**2f**)

From compound **1** (0.100 g, 0.370 mmol) and potassium (4-cyanophenyl)trifluoroborate (0.121 g, 0.592 mmol) reacting for 3 h, gave a solid which was submitted to dry flash till 90% ether/petroleum ether to give compound **2f** as a white solid (0.038 g, 35%), m.p. 198-200 °C. ^1^H-NMR (CDCl_3_, 400MHz) δ = 3.86 (3H, s, OMe), 7.44 (1H, dd, *J* = 8.0 and 4.4 Hz, 6-H), 7.65 (2H, d, *J* = 8.4 Hz, 2′ and 6′-H), 7.80 (2H, d, *J* = 8.4 Hz, 3′ and 5′-H), 8.27 (1H, broad dd, 7-H), 8.78 (1H, broad d, 5-H) ppm. ^13^C-NMR (CDCl_3_, 100.6 MHz) δ = 52.7 (OMe), 112.2 (C), 118.9 (C), 121.5 (6-CH), 131.0 (7-CH), 131.3 (2 × CH), 131.6 (2 × CH), 132.6 (C), 135.0 (C), 138.1 (C), 141.4 (C), 148.8 (5-CH), 153.4 (C), 162.2 (C=O) ppm. HRMS (EI) M^+^ Calcd. for C_16_H_10_N_2_O_2_S: 294.0463; found: 294.0474.

##### Methyl 3-(pyridin-4-yl)thieno[3,2-*b*]pyridine-2-carboxylate (**2g**)

From compound **1** (0.120 g, 0.443 mol) and 4-pyridine boronic acid (0.0790 g, 0.709 mmol), of PdCl_2_(dppf).CH_2_Cl_2_ 4 mol% and heating for 4 h, a solid was obtained and submitted to dry flash chromatography till 80% ether/petroleum ether to give compound **2g** as a white solid (0.0400 g, 66%), m.p. 192-193 °C. ^1^H-NMR (CDCl_3_, 400MHz) δ = 3.90 (3H, s, OMe), 7.50 (1H, dd, *J* = 8.4 and 4.4 Hz, 6-H), 7.90 (2H, broad d, 3′ and 5′-H), 8.32 (1H, dd, *J* = 8.4 and 1.2 Hz, 7-H), 8.79–8.82 (3H, m, 5-H and 2′ and 6′-H) ppm. ^13^C-NMR (CDCl_3_, 100.6 MHz) δ = 53.1 (OMe), 122.0 (6-CH), 127.8 (3′ and 5′-CH), 131.1 (7-CH), 134.4 (C), 135.1 (C), 137.7 (C), 139.3 (C), 143.0 (2′ and 6′-CH), 149.3 (5-CH), 152.6 (C), 161.7 (C=O) ppm. MS (ESI) *m*/*z* (%): 271.04 [M + H]^+^ (100%). Elemental Anal. calcd for C_14_H_10_N_2_O_2_S: C 62.21%, H 3.73%, N 10.36%, S 11.86%. Found: C 61.90%, H 3.69%, N 10.06%, S 11.79%.

##### Methyl 3-(furan-3-yl)thieno[3,2-*b*]pyridine-2-carboxylate (**2h**)

From compound **1** (0.120 g, 0.443 mmol), potassium 3-furanylboronic acid (0.0706 g, 0.709 mmol), PdCl_2_(dppf).CH_2_Cl_2_ 4%mol and heating for 4.5 h, a solid was obtained and submitted to dry flash chromatography till 30 % ether/petroleum ether to give compound **2h** as a white solid (0.0700 g, 52%), m.p. 130–132 °C. ^1^H-NMR (CDCl_3_, 400MHz) δ = 3.94 (3H, s, OMe), 7.02–7.03 (1H, m, HetAr-H), 7.40 (1H, dd, *J* = 8.4 and 4.4 Hz, 6-H), 7.56–7.57 (1H, m, HetAr-H), 8.21 (1H, dd, *J* = 8.4 and 1.6 Hz, 7-H), 8.25–8.26 (1H, m, HetAr-H), 8.81 (1H, dd, *J* = 4.4 and 1.6 Hz, 5-H) ppm. ^13^C-NMR (CDCl_3_, 100.6 MHz) δ = 52.6 (OMe), 112.3 (CH), 116.7 (C), 121.2 (6-CH), 130.0 (C), 130.6 (7-CH), 134.5 (C), 134.6 (C), 141.8 (CH), 144.3 (CH), 148.1 (5-CH), 153.7 (C), 162.8 (C=O) ppm. MS (ESI) *m*/*z* (%): 260.04 [M + H]^+^ (100%). Elemental Anal. calcd for C_13_H_9_NO_3_S: C 60.22%, H, 3.50%, N 5.40%, S 12.37%. Found: C 60.27%, H 3.33%, N 5.69%, S 12.48%.

### 4.2. In Vitro Antitumor Evaluation

#### 4.2.1. Reagents

Fetal bovine serum (FBS) and phosphate buffered saline (PBS) were purchased from Biowest and Gibco, respectively. Acetic acid, dimethyl sulfoxide (DMSO), ethylene diamine tetraacetic acid (EDTA), sulforhodamine B (SRB), trypan blue reagent, doxorubicin, trichloroacetic acid (TCA), Tris base and bromodeoxyuridine (BrdU) were purchased from Sigma-Aldrich. Stock solutions of the synthesized compounds were prepared with the solvent DMSO as vehicle, at stock concentration of 60 mM and kept at −20 °C. The other working stock solutions of 40 mM, 20 mM and 5 mM were also prepared in DMSO and kept at −20 °C.

#### 4.2.2. Solutions of the Compounds

Appropriate dilutions of compounds **2a–2h** were freshly prepared from the previous stock solutions in DMSO for the cell-based assays. The effect of the vehicle solvent (DMSO) on the growth of the cell lines was evaluated by exposing untreated control cells to the maximum concentration of DMSO used in each compound for each assay. 

#### 4.2.3. Cell Cultures

Two human triple negative breast cancer (TNBC) cell lines were used in this work: MDA-MD-231 and MDA-MB-468. Both TNBC cell lines were cultured in RPMI 1640 medium (Roswell Park Memorial Institute) with 25 mM of glutamine and 4-(2-Hydroxyethyl)piperazine-1-ethanesulfonic acid (HEPES) buffer (from Biowest). These media were supplemented with 5% FBS for the cell growth inhibition assay (SRB) or with 10% FBS for the remaining experiments. The non-tumorigenic cell line MCF-12A was cultured in RPMI 1640 medium supplemented with hEGF (human epidermal growth factor; 20 ng/μL), hydrocortisone (500 ng/μL), 5% FBS and 1% Penicillin-Streptomycin (Gibco), as previously described [30]. All the cell lines were maintained as adherent cell cultures and kept at 37 °C in a humidified incubator containing 5% of CO_2_. Regularly, all cells were observed using an inverted light microscope (Leica DMi1). All the experiments were carried out with cells at the exponential phase of growth and with more than 90% viability. 

#### 4.2.4. Trypan Blue Exclusion Assay

The cell number and viability were assessed by counting the cells with a hemocytometer (*Neubauer Chamber)* using the trypan blue reagent, which distinguishes alive (bright) from dead cells or non-viable cells (blue ones).

#### 4.2.5. Cell Growth Inhibition Assay (SRB Assay)

The potential cytotoxicity of the compounds was tested using an assay based on a protein-binding dye, the sulforhodamine B (SRB), according to the protocol [31]. Briefly, cells were seeded at an appropriate concentration previously optimized (5.0 × 10^4^ cells per mL for MDA-MD-231 and MDA-MB468 cells) by adding 100 μL of cells per well and then incubated at 37 °C for 24 h. All experiments were performed in two 96 well-plates, a T0 plate to be analyzed at 0 h (time of treatment with the compounds) and a T48 plate to be analyzed 48 h later. Then, cells were treated with five serial dilutions of each compound (ranging from 150 µM to 9.4 µM) by adding 100 μL of compound per well, and incubated at 37 °C for further 48 h. The effect of the vehicle (DMSO) on cell growth was also evaluated (control) by exposing untreated cells to the maximum concentration of DMSO (always lower than 0.25%). The maximum concentration tested for each compound was 150 mM. However, for some compounds, precipitation was detected within the tested range of concentrations. In those cases, the GI_50_ concentration was not possible to determine and was indicated as “higher than” the highest concentration tested without causing precipitation. Doxorubicin (ranging from 100 nM to 6.25 nM) was used as positive control. Following 48 h incubation of cells with the compounds in the T48 plate (or 0 h in the T0 plate), cells were fixed by adding 10% (*w*/*v*) of cold TCA (*w*/*v*, final concentration) during 1 h at 4 ^°^C. Subsequently, after washing with water, air-dried cells were stained with 0.4% of SRB (in acetic acid, *w*/*v*) for 30 min in the dark at room temperature (RT). At the end, cells were washed with 1% (*v*/*v*) of acetic acid and the bound dye was solubilized with 10 mM Tris base solution. The absorbance was measured at 510 nm in a microplate reader (BioTek’s SynergyTM Mx) using the Gen5 software (BioTek) [19,20]. The GI_50_ concentrations for each compound (concentrations that inhibited cell growth by 50%) were assessed from the dose-response curves, determined in each cell line and presented as mean ± standard error (S.E.M) from at least three independent experiments. 

Compounds **2e**, **2f**, **2h** were also tested against the non-malignant MCF-12A human breast epithelial cells. For that, cells (seed at 5.0 × 10^4^ cells per mL) were incubated with the GI_50_ concentrations of each of the three compounds obtained in the corresponding tumor cell lines for 48 h, followed by removal of the compound, addition of new medium to the cells and then 5 more days in culture. At the end of the 7 days in total, the sulforhodamine B (SRB) assay was performed, as previously described [30].

#### 4.2.6. Cell Proliferation Analysis (BrdU Assay)

Cell proliferation was assessed using the bromodeoxyuridine (BrdU) incorporation assay, according to the previously described protocol [19]. The MDA-MB-231 cells were plated at 7 × 10^4^ cells per mL in 6-well plates. After 24 h, cells were treated with the GI_50_ (13 μM) of compound **2e**, medium (blank), the vehicle corresponding to DMSO (control; in a concentration equivalent to the one used in the treatment) or 50 nM of doxorubicin (positive control), for 48 h. After that, cells were incubated with 10 µM BrdU (Sigma-Aldrich) for 4 h. Cells were washed and fixed with 4% paraformaldehyde, PFA (Panreac) in PBS. After the preparation of cytospins, cells were treated with 2 M HCl for 20 min (DNA denaturation), followed by blocking with a PBS 0.5% Tween and 0.05% Bovine Serum Albumin solution. Cells were then incubated with a mouse anti-BrdU antibody (1:10; Dako), followed by the incubation with anti-mouse-Ig-FITC (1:100; Dako). After that, slides were prepared with Vectashield mounting medium containing 4′,6-diamidino-2-phenylindole, DAPI (Vector Laboratories Inc, Burlingame, CA, USA). The detection of BrdU incorporation (green nuclei) was possible using a Zeiss Axio Imager Z1 (Carl Zeiss, Jena, Germany) microscope and the Axiovision 4.9 (Carl Zeiss, Jena, Germany) software. A semi-quantitative evaluation was performed by counting a minimum of 500 cells per slide using the ImageJ 2.1.0 software. The results were from three independent experiments, and are presented as mean ± S.E.M.

#### 4.2.7. Cell Cycle Analysis

For cell cycle distribution analysis, MDA-MB-231 cells were plated at 7 × 10^4^ cells per mL in 6-well plates for 24 h. Cells were then treated with the GI_50_ (13 μM) of the compound **2e**, medium (blank), the vehicle DMSO (control) and 50 nM of doxorubicin (positive control). Following 48 h incubation, cells were collected, centrifuged (1200 rpm for 5 min) and then fixed with ice-cold 70% ethanol and stored at 4 °C for at least 12 h. Cells were then centrifuged at 1200 rpm for 5 min, at 4 °C, and the pellets resuspended in a solution of PBS containing 5 μg/mL propidium iodide (PI) and 0.1 mg/mL RNase A, and incubated for at least 30 min in the dark on ice, prior to each analysis on the flow cytometer. Cellular DNA content for cell cycle distribution was analyzed by flow cytometry using the BD Accuri™ C6 Flow Cytometer (BD Biosciences). The exclusion of cell debris and aggregates was performed for each analysis, and at least 20,000 events per sample were plotted for all the acquisitions. Data was analyzed using the FlowJo software (version 7.6.5, Tree Star, Inc., Ashland, OR, USA) [19]. The results were from at least three independent experiments, and are presented as mean ± S.E.M.

### 4.3. In Ovo Evaluation

#### In Vivo Chick Egg Chorioallantoic Membrane (CAM) Model

The chick embryo chorioallantoic membrane (CAM) model was used to evaluate the tumorigenic response of compound **2e**. Fertilized chick (*Gallus gallus*) eggs were obtained from commercial sources, and incubated horizontally at 37.8 °C, in a humidified atmosphere and referred to the embryonic development day (EDD). Ethical approval is not required for experiments using embryonic chicken according to the European Directive 2010/63/EU and the Portuguese law on animal welfare does not restrict the use of chicken eggs for experimental purposes. Briefly, on EDD3, a square window was opened in the shell after removal of 1.5–2 mL of albumin to allow detachment of the developing CAM. The window was sealed with a transparent adhesive tape and the eggs returned to the incubator. The MDA-MB-231 cells were resuspended in matrigel (1:1) and grafted on top of the same EDD9 growing CAM, into two independent 3-mm nylon rings, under sterile conditions (7.5 × 10^5^ cells per ring). The eggs were resealed and returned to the incubator for an additional 24 h. At EDD10, tumor xenografts were treated with either vehicle (DMSO) or with compound **2e** pairwise. Eggs were resealed and returned to the incubator for additional 3 days. On EDD14, embryos were euthanized by adding 2 mL of fixative in the top of the CAM, rings were removed, the CAM was excised from embryos and photographed ex-ovo under a stereoscope, using a 20× magnification (Olympus, SZX16 coupled with a DP71 camera). The area of CAM tumors was determined using the Cell A (Olympus) software. The experiment was performed in a blind fashion way, since the identity of the treatment (vehicle or compound **2e**) was not known by the person performing the analysis. The area of CAM tumors was determined using the Olympus cell Sens Standard 1.14 program. Excised CAMs were fixed in 10% neutral-buffered formalin, paraffin-embedded for slide sections and stained with hematoxylin–eosin for histological examination.

### 4.4. Statistical Analysis

The statistical analysis was performed using GraphPad Prism Software v5 (GraphPad-trial version, San Diego, CA, USA). For SRB assay, the GI_50_ concentrations were assessed from the dose-response curves and determined for each compound, in each cell line. The GI_50_ values presented are the mean ± standard error (S.E.M.) from at least three independent experiments. For BrdU and cell cycle analysis, the % of proliferating cells or cells in each cell cycle phase was determined, and the results presented are the mean ± S.E.M. of at least three independent experiments. In the CAM assay, the data (*n* = 19) were analyzed for Gaussian distribution and passed the D’Agostino and Pearson normality tests. To test the hypothesis that compound **2e** treated tumors are different from the vehicle, a paired t-test was used. Statistical significance was achieved when *p* value < 0.05. 

## 5. Conclusions

Eight novel methyl 3-(hetero)arylthieno[3,2-*b*]pyridine-2-carboxylates **2a**–**2h** were prepared in moderate to high yields by C-C Pd-catalyzed Suzuki-Miyaura cross-coupling of methyl 3-bromothieno[3,2-*b*]pyridine-2-carboxylate with different boronated compounds. These were tested against TNBC cell lines and the potential of three novel thieno[3,2-*b*]pyridine derivatives **2e**, **2f**, **2h** as anticancer agents in TNBC cell lines was demonstrated. Importantly, no toxicity was observed in non-tumorigenic breast cells. One of the most promising compounds, **2e**, presented activity against TNBC both in vitro (by decreasing viable cell number and cell proliferation and interfering with the cell cycle profile) and in ovo *(*by reducing grafted tumor size). However, further studies must be carried out to unravel the underlying mechanisms of action of this compound. Furthermore, the anticancer effect of compound **2e** should be also assessed in vivo, using xenografted mouse models of human TNBC cell lines. Although the present work is focused on compound **2e**, the other promising compounds (**2f** and **2h**) will be also explored in the future. Therefore, this work highlights the antitumor potential of a novel thieno[3,2-*b*]pyridine derivative against an aggressive type of cancer, TNBC. 

## Figures and Tables

**Table 1:** Synthesis of methyl 3-(hetero)arylthieno[3,2-*b*]pyridine-2-carboxylates **2a**–**2h** products.

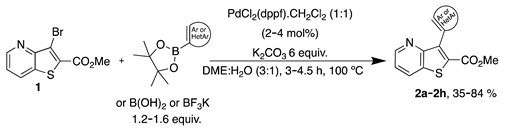			
Boronated Compounds	Suzuki-Miyaura Products, Yields and Reaction Times *	Boronated Compounds	Suzuki-Miyaura Products, Yields and Reaction Times *
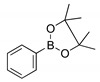	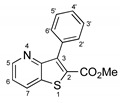 **2a**, 74%, 3 h	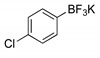	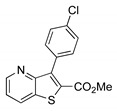 **2e**, 82%, 3 h
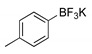	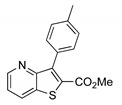 **2b**, 84%, 4.5 h	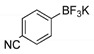	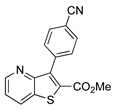 **2f**, 35%, 3 h
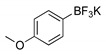	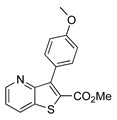 **2c**, 70%, 4 h	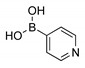	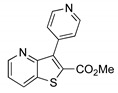 **2g**, 66%, 4 h
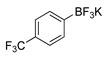	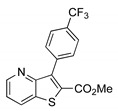 **2d**, 40%, 4 h	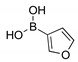	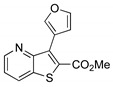 **2h**, 52%, 4.5 h

* The products yields were calculated after column chromatography.

**Table 2:** GI_50_ concentrations of compounds **2a**–**2h** in two different triple negative breast cancer cells, determined with SRB assay.

GI_50_ * Concentrations (µM) for Each Cancer Cell Line		
Compound (μM) Treatment for 48 h	MDA-MB-231	MDA-MB-468
**2a**	>20	>20
**2b**	>10	>10
**2c**	>10	>10
**2d**	>10	>30
**2e**	12.56 ± 1.88	>14
**2f**	28.67 ± 1.34	8.73 ± 1.73
**2g**	>75	>75
**2h**	>75	4.67 ± 0.68
Doxorubicin	0.068 ± 0.006	0.081 ± 0.009

* GI_50_ values correspond to the mean ± S.E.M. of at least three independent experiments, all performed in duplicated. When not possible to determine the GI_50_ due to the formation of crystals, the highest concentration tested in which crystals were not observed is presented (GI_50_ higher than that concentration).

**Table 3:** Evaluation of the cytotoxicity of the three most promising compounds in the non-tumorigenic cell line MCF-12A, using the SRB assay.

Compounds	Concentrations Tested (µM) (GI_50_ Concentration in the Tumor Cell Lines)	% Cell Growth in MCF-12A Cell Line (Relative to the Control) *
**2e**	12.56	88.62 ± 4.04
**2f**	8.73	117.73 ± 3.22
**2h**	4.67	82.13 ± 4.78

* Values correspond to the mean ± S.E.M. of at least three independent experiments, all performed in duplicate. The % of growth of the cell line MCF-12A was determined using the GI_50_ concentrations of each compound, in the most sensitive tumor cell lines previously determined (Table 2).

## Data Availability

The data presented in this study are available in article, including in the Supplementary Materials.

## Supplementary Materials

^1^H, ^13^C and ^19^F NMR spectra of the novel compounds **2a**–**2h** are presented.

## Sample Availability

Not available.
