# Supplementary material for: Synthesis of Novel Methyl 3-(hetero)arylthieno[3,2-b]pyridine-2-carboxylates and Antitumor Activity Evaluation: Studies In Vitro and In Ovo Grafts of Chick Chorioallantoic Membrane (CAM) with a Triple Negative Breast Cancer Cell Line
Source: Molecules. 2021 Mar 13;26(6):1594. doi: 10.3390/molecules26061594 (PMC7999514; doi:10.3390/molecules26061594)

# Synthesis of novel methyl 3-(hetero)arylthieno[3,2-*b*]pyridine-2-carboxylates and antitumor activity evaluation: studies *in vitro* and *in ovo grafts of* Chick Chorioallantoic Membrane (CAM) with a triple negative breast cancer cell line

**Bruna R. Silva [1,2,3,#], Rita Rebelo [2,3,#], Juliana M. Rodrigues [1], Cristina P. R. Xavier [2,3*], M. Helena Vasconcelos [2,3,4], Maria-João R. P. Queiroz [1*]**

1 Centre of Chemistry, University of Minho, 4710-057, Braga, Portugal; mjrpq@quimica.uminho.pt (MJQ); brunadansilva1967@gmail.com (BS); id7018@alunos.uminho.pt (JMR)
2 Cancer Drug Resistance Group, IPATIMUP - Institute of Molecular Pathology and Immunology, University of Porto, Portugal, 4200-135, Porto, Portugal; rrebelo@ipatimup.pt (RR); cristinax@ipatimup.pt (CPRX); hvasconcelos@ipatimup.pt (MHV)
3 i3S - Instituto de Investigação e Inovação em Saúde, Universidade do Porto, 4200-135 Porto, Portugal,
4 Department of Biological Sciences, FFUP - Faculty of Pharmacy of the University of Porto, Porto, Portugal;

* Correspondence: mjrpq@quimica.uminho.pt (MJRPQ); cristinax@ipatimup.pt (CPRX)
# These authors equally contributed to this work.

## Table of contents

$^1$H and $^{13}$C NMR spectra of compound **2a**....................S2
$^1$H and $^{13}$C NMR spectra of compound **2b**....................S3
$^1$H and $^{13}$C NMR spectra of compound **2c**....................S4
$^1$H, $^{13}$C NMR and $^{19}$F spectra of compound **2d**....................S5
$^1$H and $^{13}$C NMR spectra of compound **2e**....................S7
$^1$H and $^{13}$C NMR spectra of compound **2f**....................S8
$^1$H and $^{13}$C NMR spectra of compound **2g**....................S9
$^1$H and $^{13}$C NMR spectra of compound **2h**....................S10

**Methyl 3-phenylthieno[3,2-*b*]pyridine-2-carboxylate (2a)**

$^{1}$H NMR (DMSO-$d_6$, 400 MHz) of compound **2a**

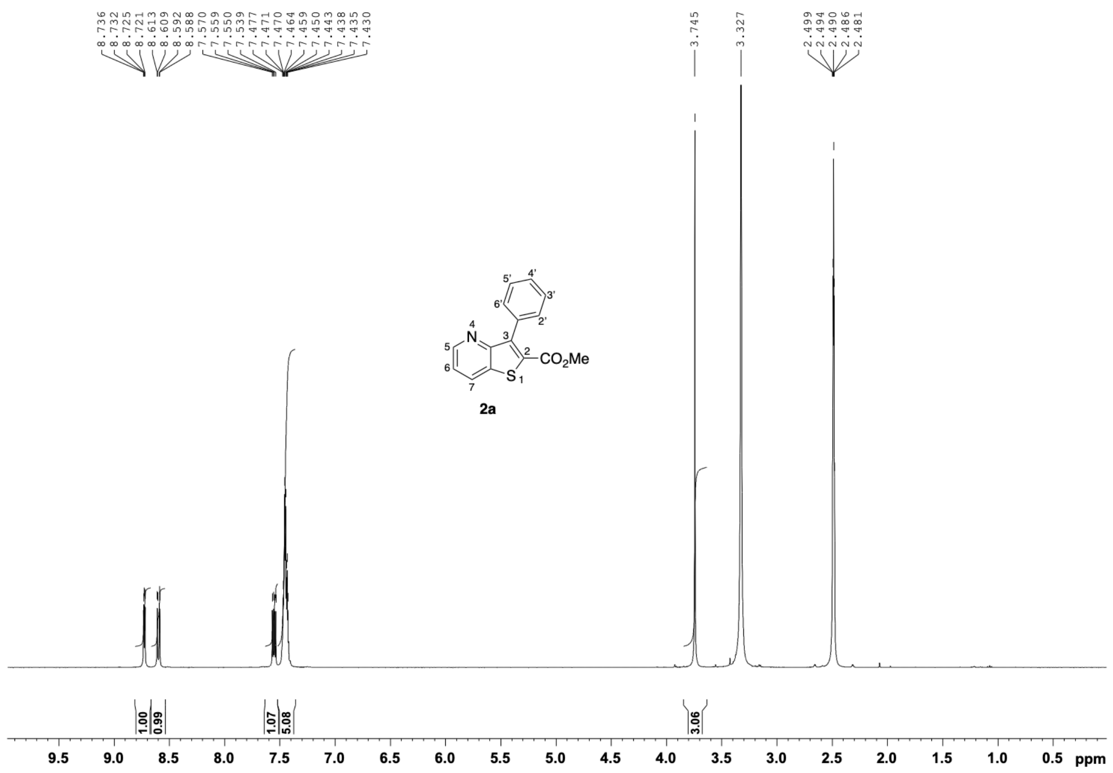


$^{13}$C NMR (DMSO-$d_6$, 100.6 MHz) of compound **2a**

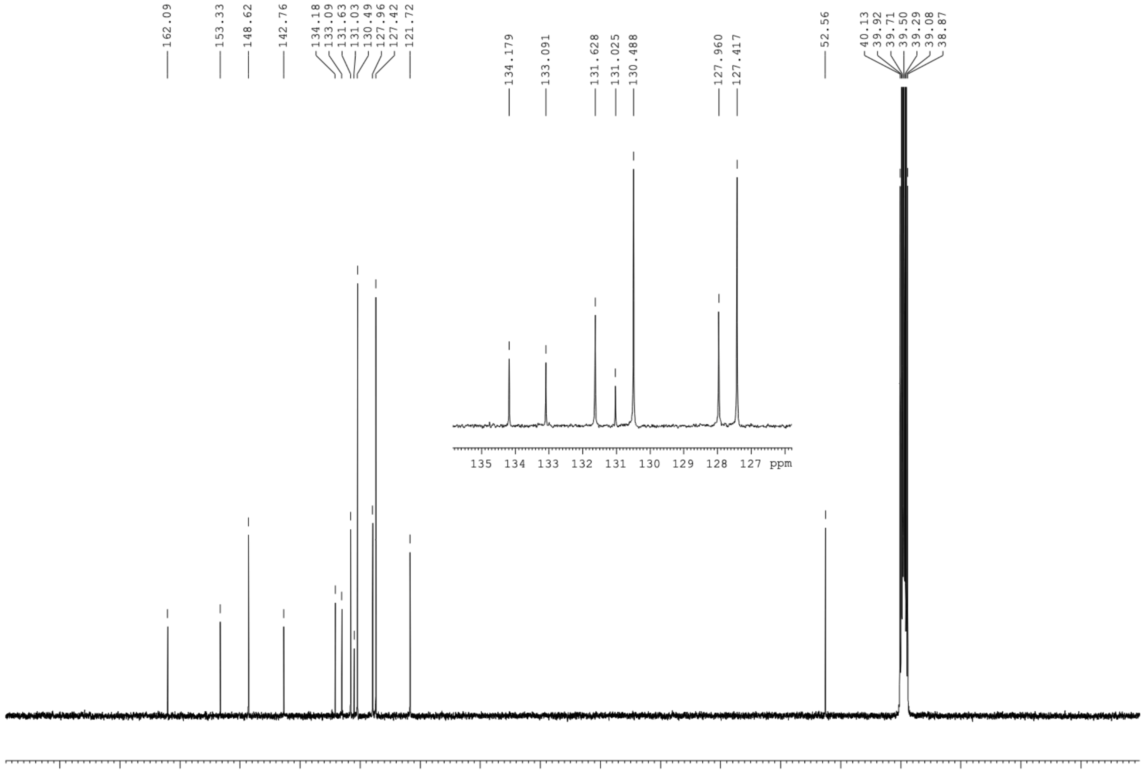

**Methyl 3-(*p*-tolyl)thieno[3,2-*b*]pyridine-2-carboxylate (2b)**

$^{1}H$ NMR ($CDCl_3$, 400 MHz) of compound **2b**

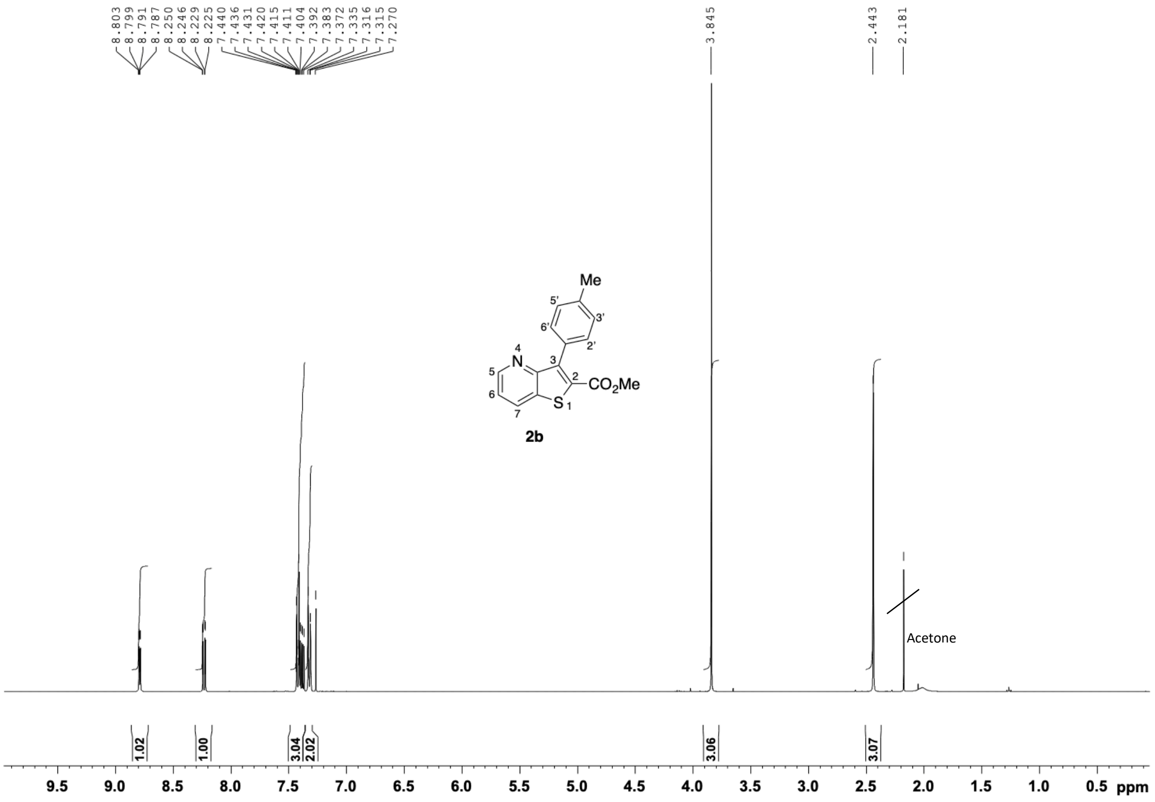


$^{13}C$ NMR ($CDCl_3$, 100.6 MHz) of compound **2b**

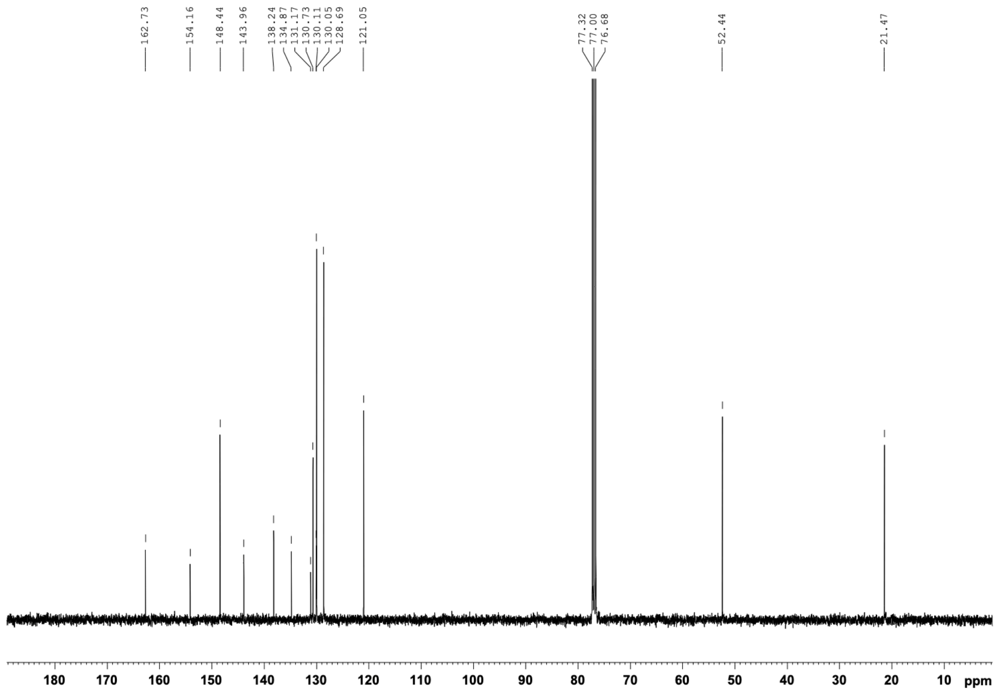

**Methyl 3-(4-methoxyphenyl)thieno[3,2-*b*]pyridine-2-carboxylate (2c)**

$^1$H NMR (CDCl$_3$, 400 MHz) of compound **2c**

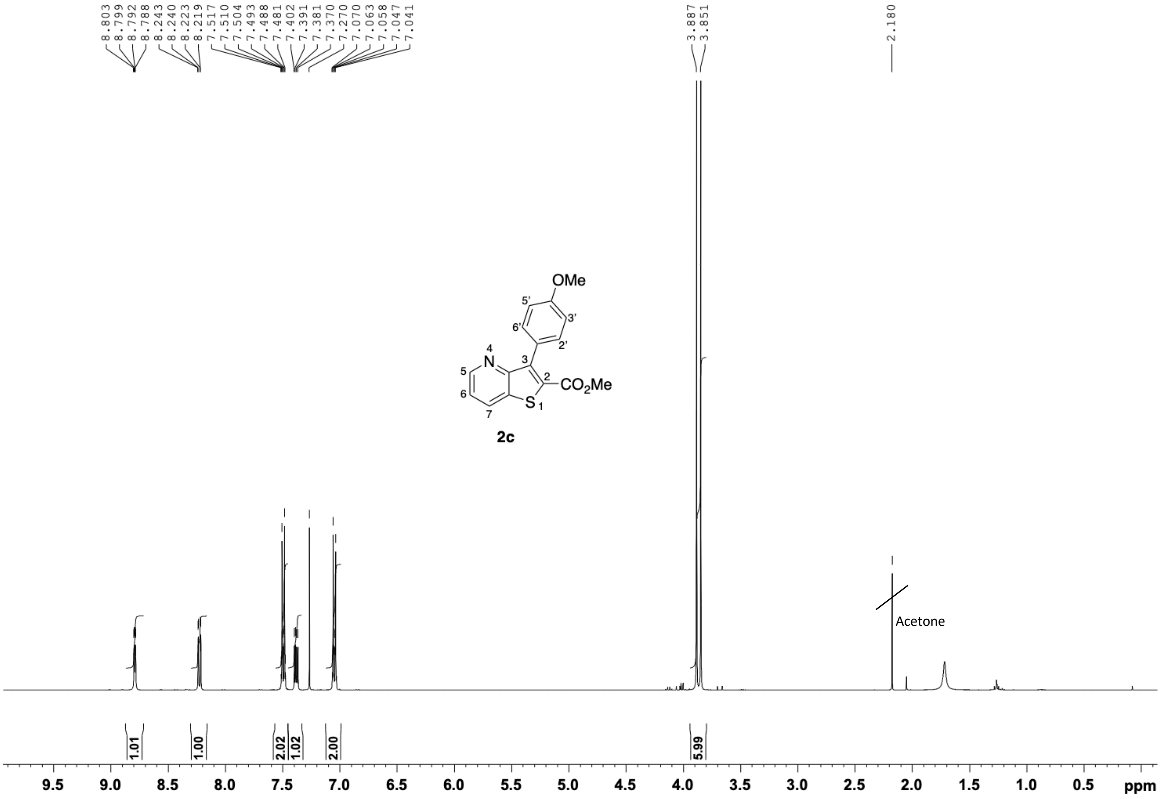


$^{13}$C NMR (CDCl$_3$, 100.6 MHz) of compound **2c**

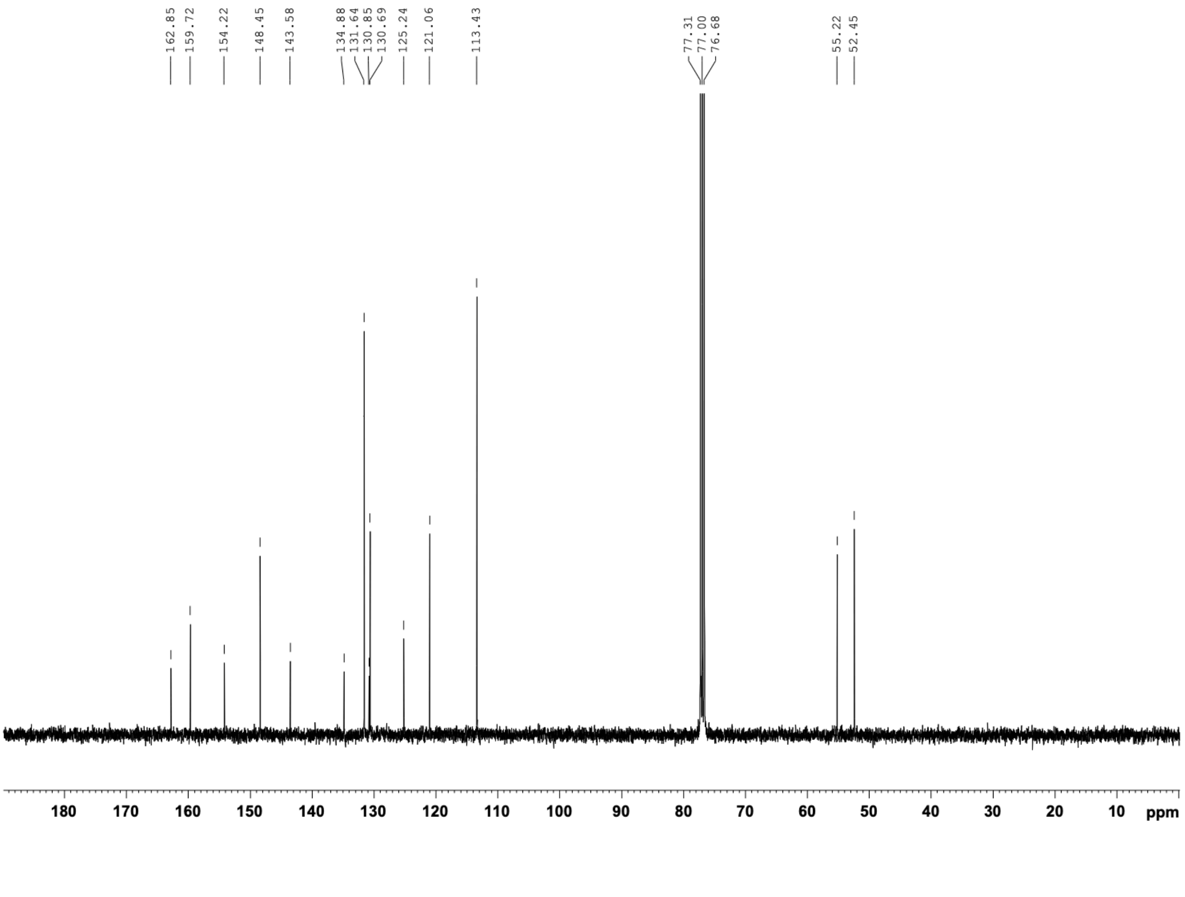

**Methyl 3-[4-(trifluoromethyl)phenyl]thieno[3,2-*b*]pyridine-2-carboxylate (2d)**

$^{1}H$ NMR ($CDCl_3$, 400 MHz) of compound **2d**

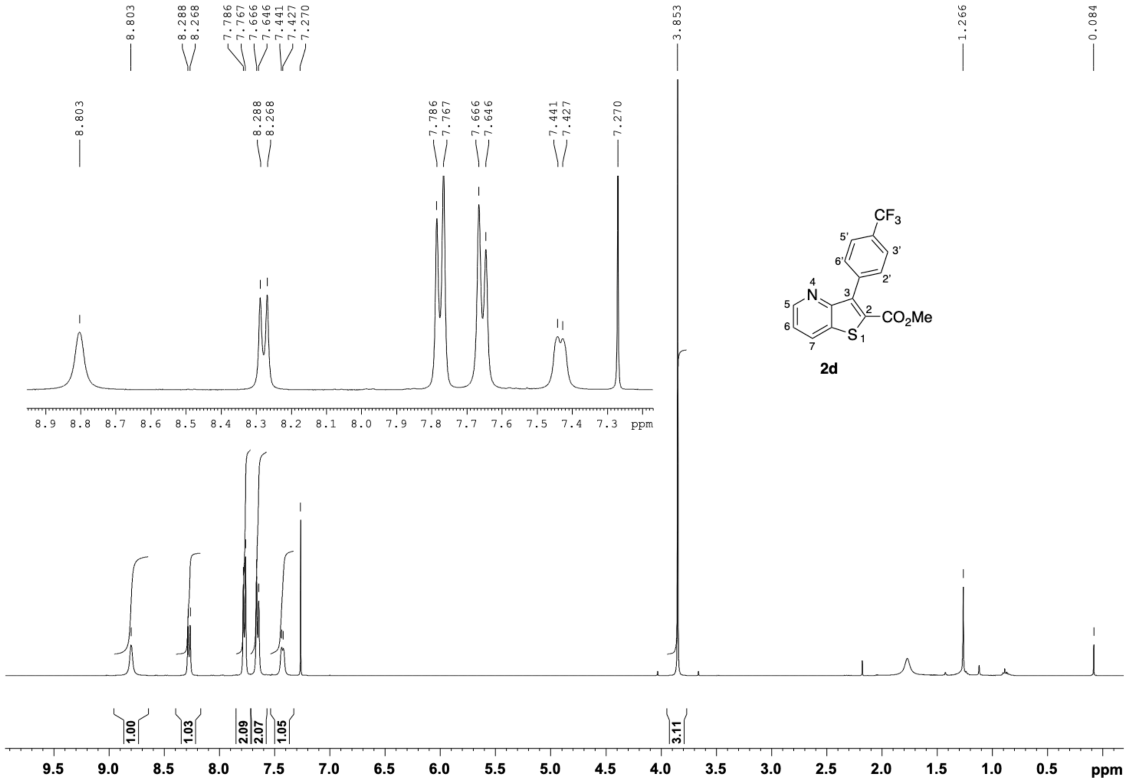


$^{13}C$ NMR ($CDCl_3$, 100.6 MHz) of compound **2d**

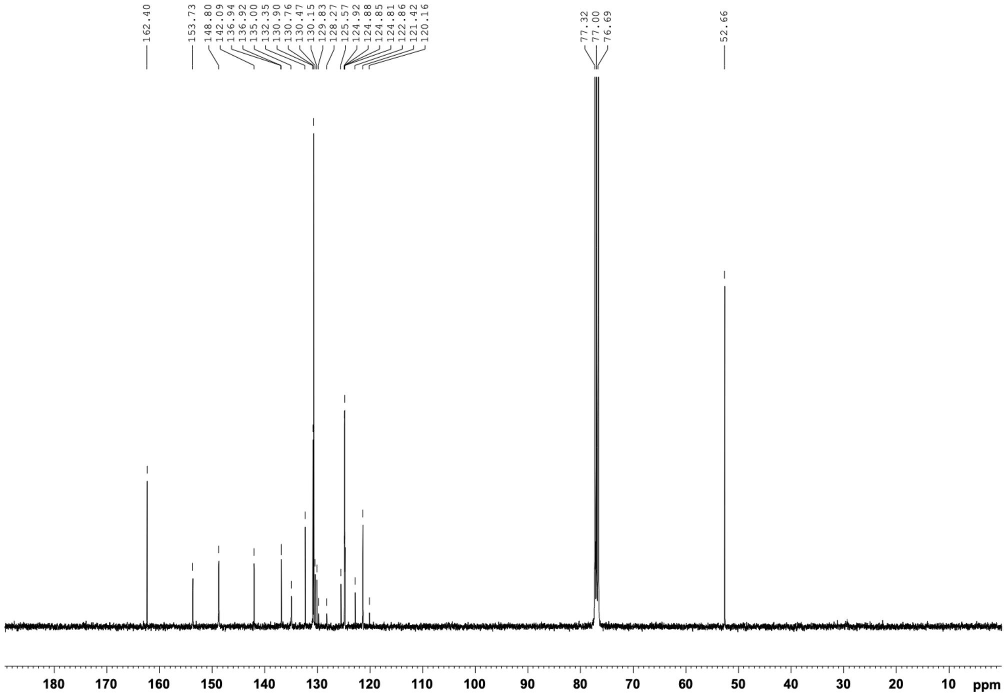

$^{19}F$ NMR ($CDCl_3$, 376.48 MHz) of compound **2d**

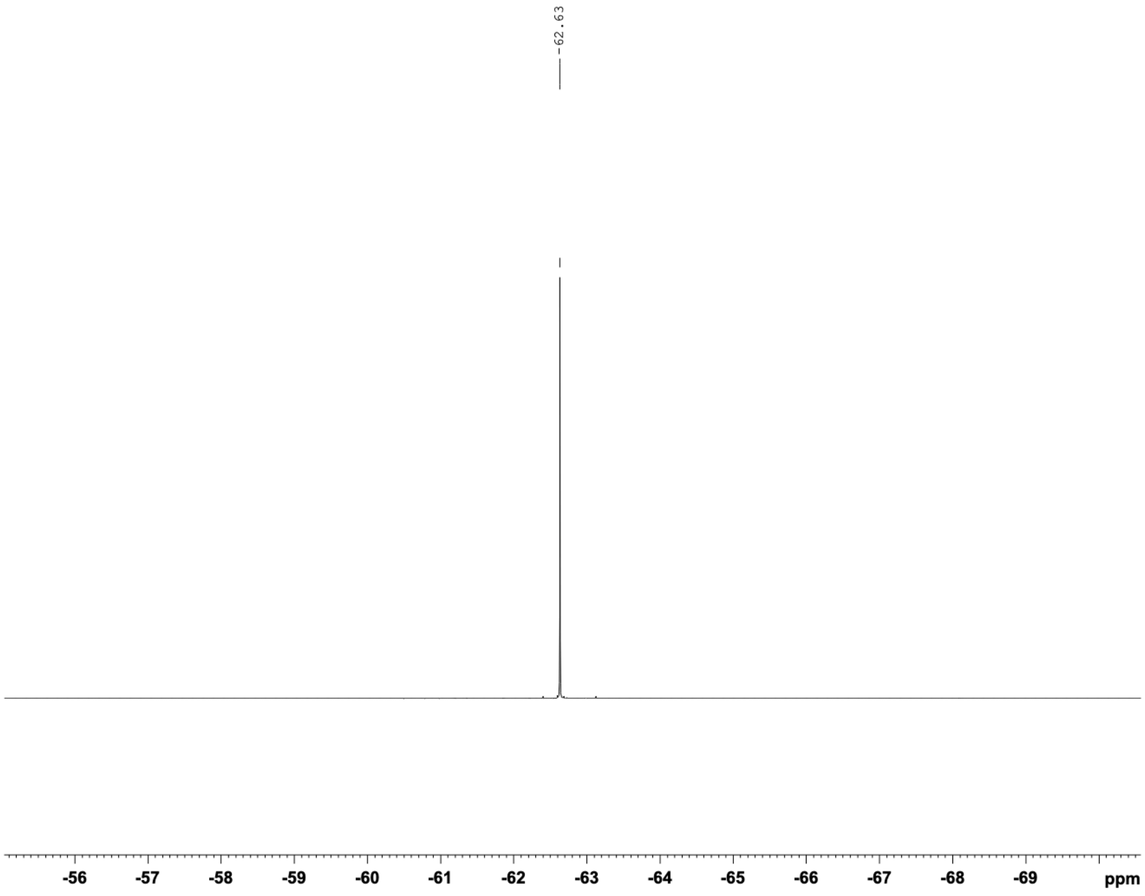

**Methyl 3-(4-chlorophenyl)thieno[3,2-*b*]pyridine-2-carboxylate (2e)**

$^{1}H$ NMR ($CDCl_3$, 400 MHz) of compound **2e**

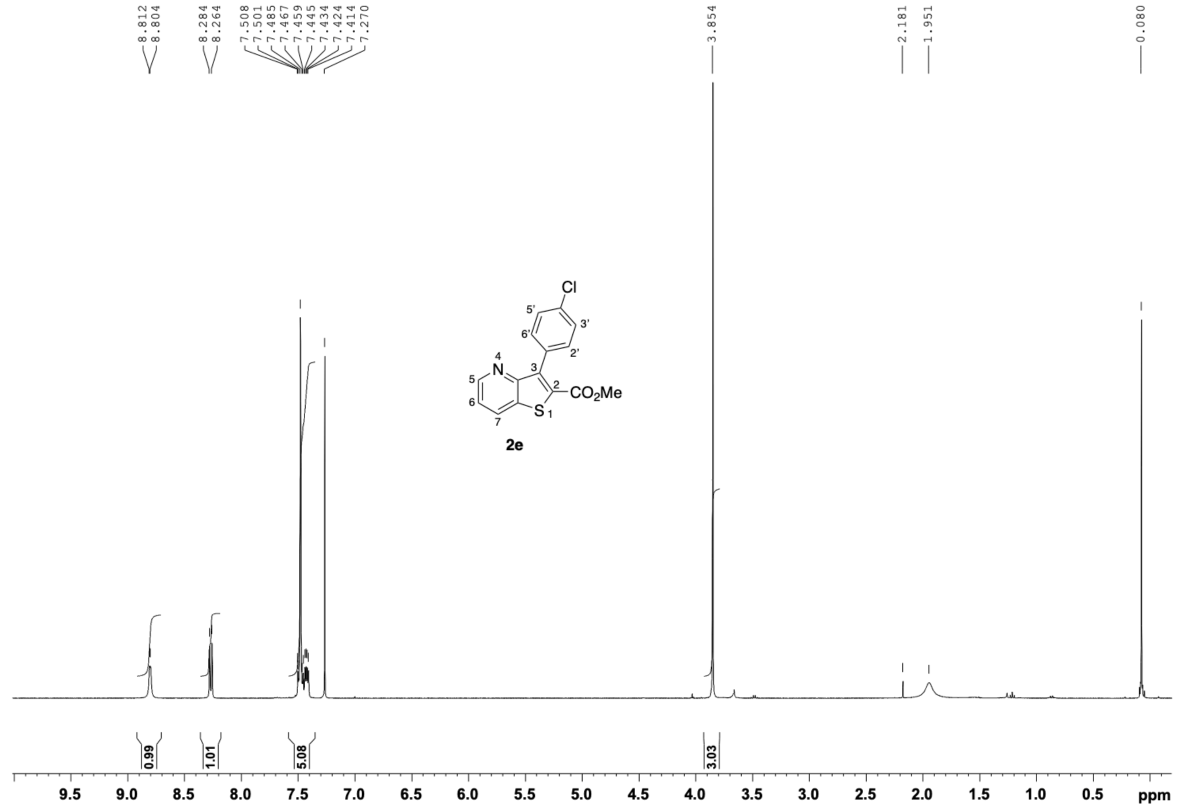


$^{13}C$ NMR ($CDCl_3$, 100.6 MHz) of compound **2e**

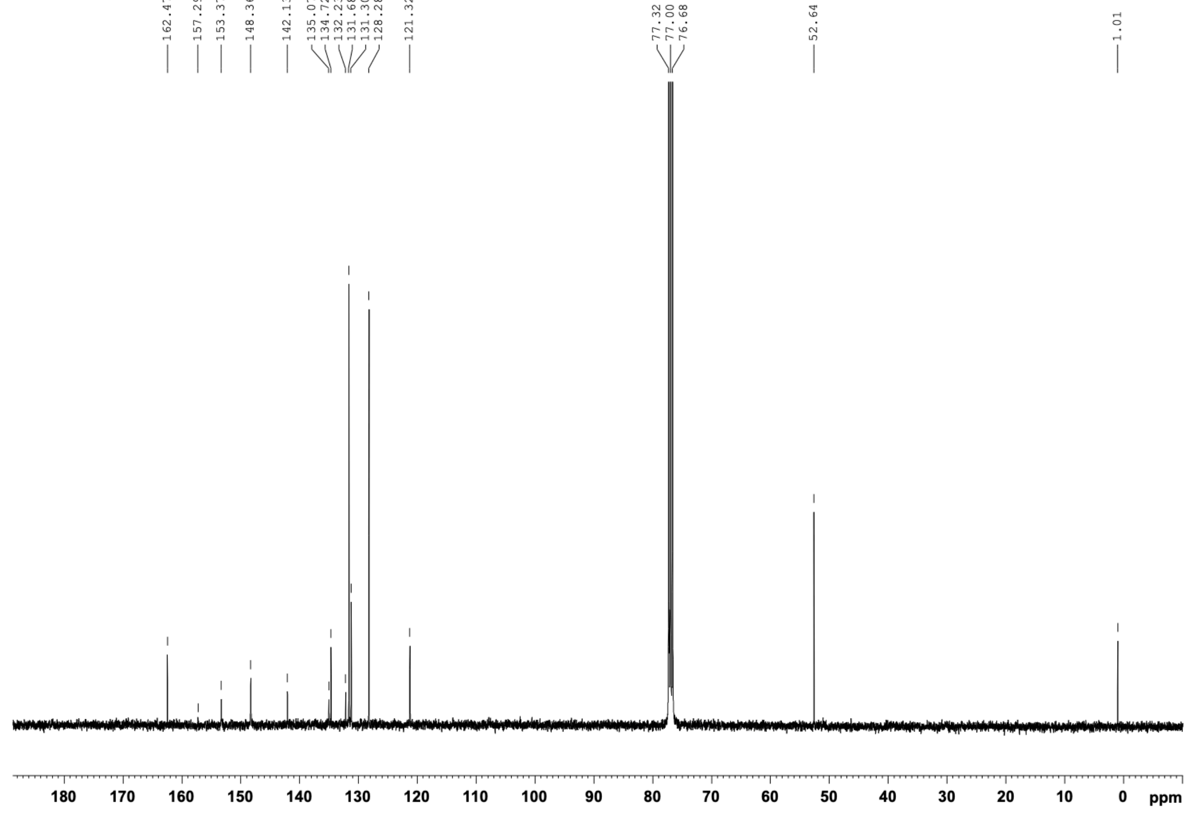

**Methyl 3-(4-cyanophenyl)thieno[3,2-*b*]pyridine-2-carboxylate (2f)**

$^{1}$H NMR ($CDCl_3$, 400 MHz) of compound **2f**

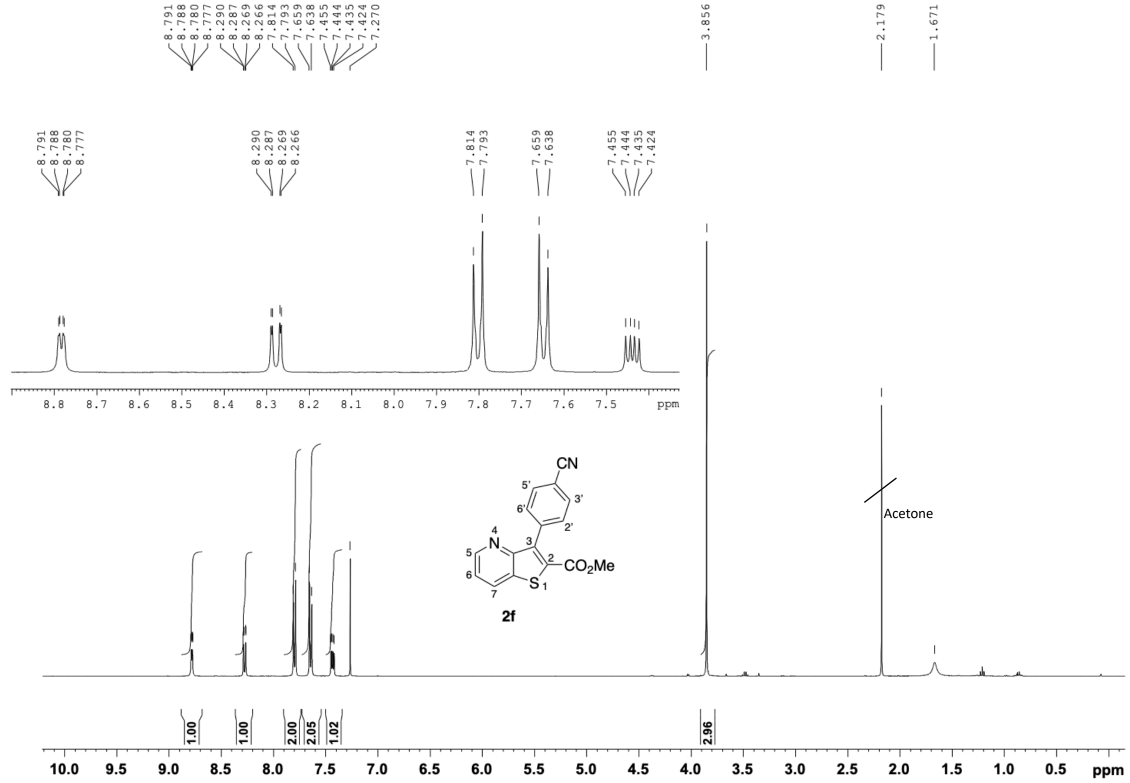


$^{13}$C NMR ($CDCl_3$, 100.6 MHz) of compound **2f**

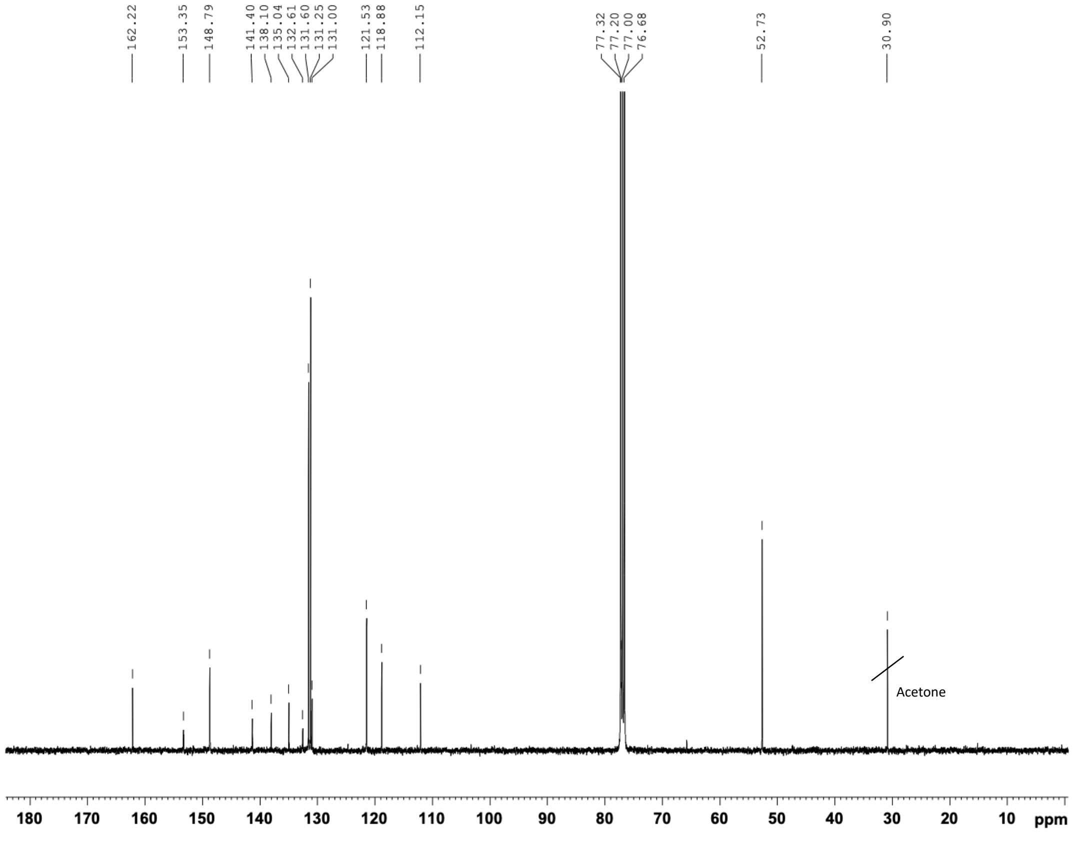

**Methyl 3-(pyridin-4-yl)thieno[3,2-*b*]pyridine-2-carboxylate (2g)**

$^{1}$H NMR ($CDCl_3$, 400 MHz) of compound **2g**

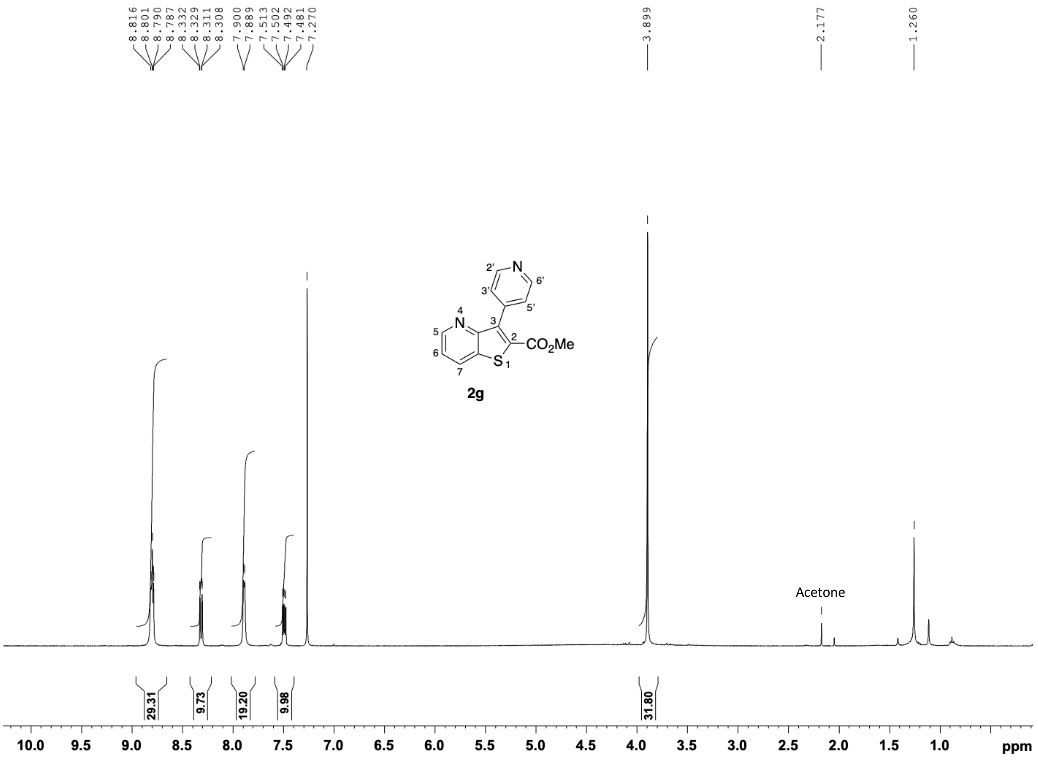


$^{13}$C NMR ($CDCl_3$, 100.6 MHz) of compound **2g**

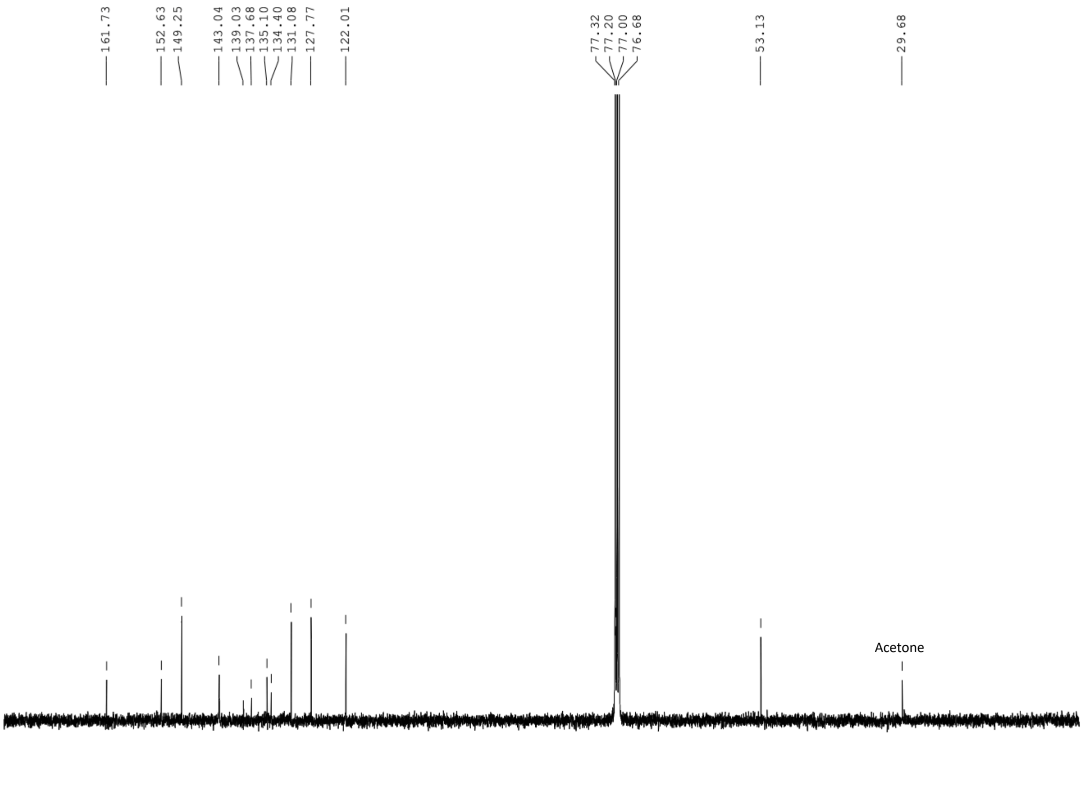


**Methyl 3-(furan-3-yl)thieno[3,2-*b*]pyridine-2-carboxylate (2h)**

$^{1}H$ NMR ($CDCl_3$, 400 MHz) of compound **2h**

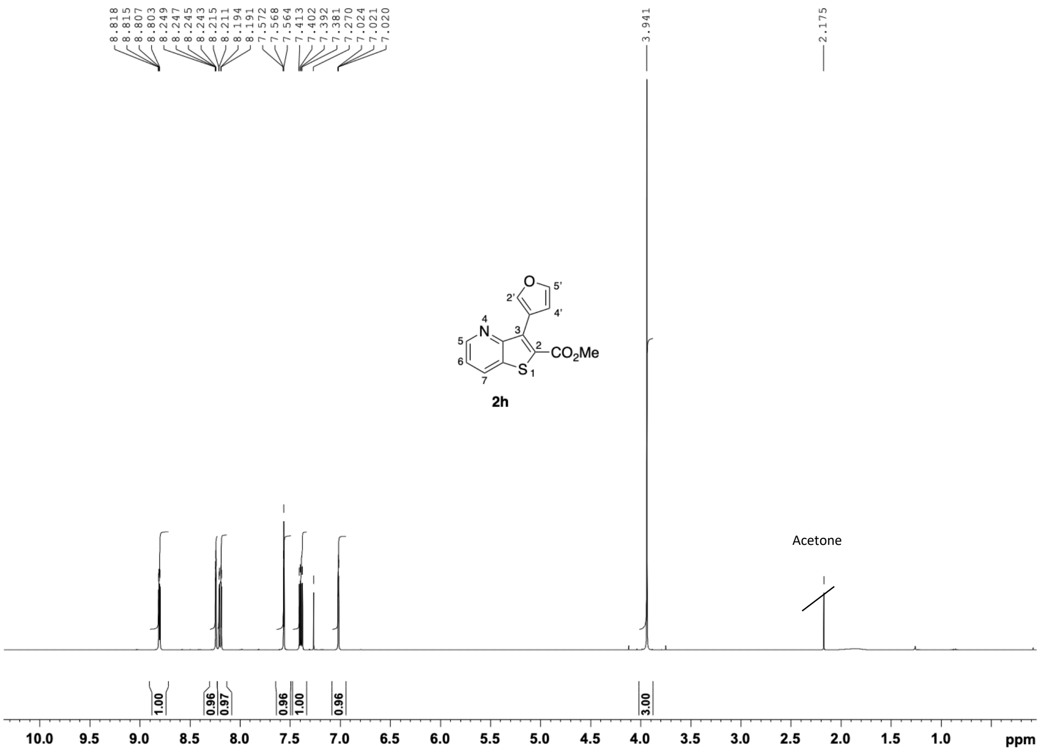


$^{13}C$ NMR ($CDCl_3$, 100.6 MHz) of compound **2h**

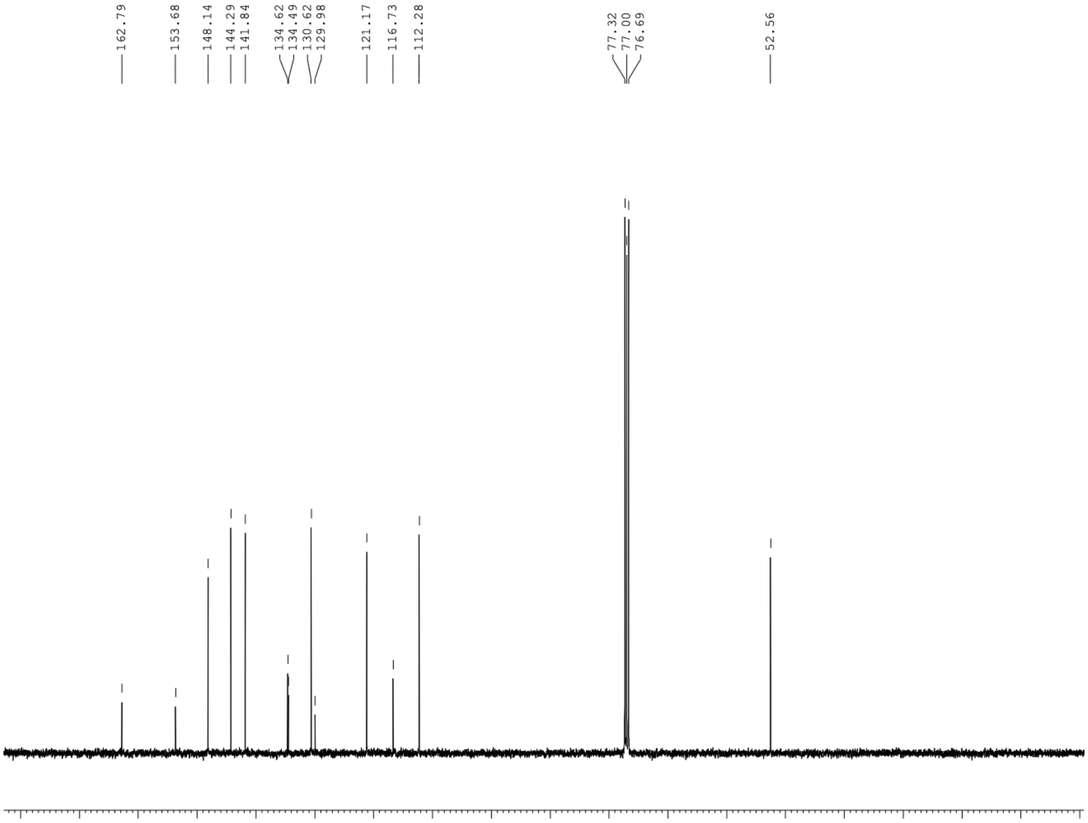

Supplement: Supplementary file 1 [file molecules-26-01594-s001.pdf]
